# Analysis of cell–nanoparticle interactions and imaging of *in vitro* labeled cells showing barcorded endosomes using fluorescent thiol-organosilica nanoparticles surface-functionalized with polyethyleneimine†

**DOI:** 10.1039/d1na00839k

**Published:** 2022-05-06

**Authors:** Michihiro Nakamura, Junna Nakamura, Chihiro Mochizuki, Chika Kuroda, Shigeki Kato, Tomohiro Haruta, Mayu Kakefuda, Shun Sato, Fuyuhiko Tamanoi, Norihiro Sugino

**Affiliations:** Department of Organ Anatomy and Nanomedicine, Graduate School of Medicine, Yamaguchi University 1-1-1 Minami-Kogushi Ube Yamaguchi 755-8505 Japan nakam@yamaguchi-u.ac.jp; EM Application Group, EM Business Unit, JEOL Ltd. Japan; Department of Obstetrics and Gynecology, Graduate School of Medicine, Yamaguchi University 1-1-1 Minami-Kogushi Ube Yamaguchi 755-8505 Japan; Department of Microbiology, Immunology and Molecular Genetics, University of California Los Angeles CA 90095 USA; Institute for Integrated Cell-Material Sciences, Institute for Advanced Study, Kyoto University Yoshida-Honmachi, Sakyo-ku Kyoto 606-8501 Japan

## Abstract

Biomedical imaging using cell labeling is an important technique to visualize cell dynamics in the body. To label cells, thiol-organosilica nanoparticles (thiol-OS) containing fluorescein (thiol-OS/Flu) and rhodamine B (thiol-OS/Rho) were surface-functionalized with polyethyleneimine (PEI) (OS/Flu-PEI and OS/Rho-PEI) with 4 molecular weights (MWs). We hypothesized PEI structures such as brush, bent brush, bent lie-down, and coiled types on the surface depending on MWs based on dynamic light scattering and thermal gravimetric analyses. The labeling efficacy of OS/Flu-PEIs was dependent on the PEI MW and the cell type. A dual-particle administration study using thiol-OS and OS-PEIs revealed differential endosomal sorting of the particles depending on the surface of the NPs. The endosomes in the labeled cells using OS/Flu-PEI and thiol-OS/Rho revealed various patterns of fluorescence termed barcoded endosomes. The cells labeled with OS-PEI *in vitro* were administrated to mice intraperitoneally after *in situ* labeling of peritoneal cells using thiol-OS/Rho. The *in vitro* labeled cells were detected and identified in cell aggregates *in vivo* seamlessly. The labeled cells with barcoded endosomes were also identified in cell aggregates. Biomedical imaging of *in vitro* OS-PEI-labeled cells combined with *in situ* labeled cells showed high potential for observation of cell dynamics.

## Abstract 2

“Endosomal barcoded cells” *via* the differential intraendosomal sorting of particles depending on the particle surface were prepared using simultaneous dual-particle administration and observed on seamless biomedical imaging.

## Graphical abstract

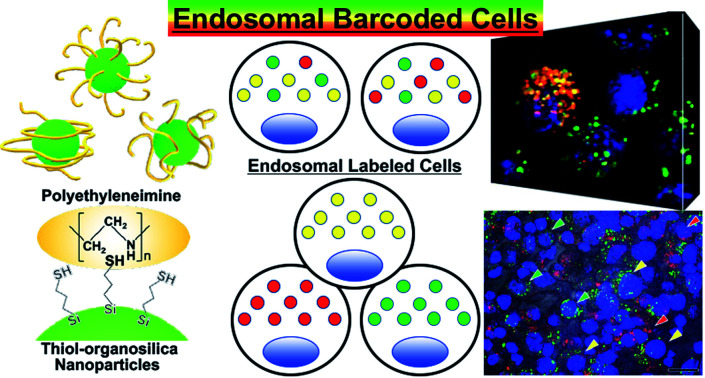

## Introduction

Biomedical imaging using cell labeling is an important technique to observe the localization, migration, and function of cells in tissues, organs, and the body. Cell labeling has been applied in various research fields, such as immunology, neuroscience, developmental biology, regenerative biology, and oncology. Various materials and techniques have been developed to label cells efficiently and effectively for observation. Recently, transgene labeling using the reporter genes of fluorescent proteins has become very popular in various fields.^1,2^ Similar to other techniques, fluorescent dyes such as PKH-26 were applied for cell labeling *via* their nonspecific affinity to cells (passive labeling).^3,4^ To label cells specifically, fluorescent probes were conjugated with high-affinity and specific ligands, such as antibodies, and the ligand-conjugated probes were applied to cell labeling (active labeling) and imaging.^5–7^ The main imaging modality of cells labeled *via* passive and active labeling is optical imaging. Optical imaging has some advantages over other modalities, such as high sensitivity and temporal resolution, for observing labeled cells *in vitro* as well as *in vivo*. The new trend of cell labeling techniques advances with the addition and expansion of other imaging modalities such as X-ray computerized tomography and magnetic resonance imaging. This trend has been driven by the application of multifunctional nanoparticles (NPs).^8–12^ Multifunctional NPs have provided new potential for cell labeling and imaging because of their advantageous properties. Gold NPs and iron oxide NPs were applied to other modalities, such as X-ray computerized tomography and magnetic resonance imaging (MRI) with higher spatial resolution and 3-dimensional (3-D) capabilities, which overcome the limitations of optical imaging.

We have prepared and reported various types of fluorescent organosilica NPs.^13,14^ Organosilica particles differ both structurally and functionally from typical inorganosilica particles prepared from tetraethoxyorthosilicate. Thiol-organosilica particles are prepared from 3-mercaptopropyltrimethoxysilane (MPMS) as a single organosilicate source and contain both interior and exterior functionalities as prepared. The organosilica particles can be internally functionalized with various fluorescent molecules and nanomaterials simply and easily. The mercaptopropyl residues on their surface are useful for functionalization, and can bind with biomolecules *via* various interactions such as electrostatic interactions, hydrophobic interactions, and hydrogen bonds. Fluorescent thiol-organosilica particles (thiol-OS) were useful for various fluorescence imaging techniques, such as fluorescence *in vivo* and *in vitro* cell imaging.^13,15^ Single-cell imaging and analysis of the peritoneal macrophage uptake of NPs revealed heterogeneity of uptake and could identify polyethylene-glycol-resistant macrophages.^16,17^

This paper reports the applications of thiol-OS surface-functionalized with the positively charged polymer polyethyleneimine (PEI) (OS-PEIs) with 4 molecular weights (MWs) to label cells for imaging. We characterized OS-PEIs and proposed their novel model depending on the surface structure. And then we performed cell–nanoparticle interaction analysis using 4 kinds of cells and 4 kinds of thiol-OS containing fluorescein (thiol-OS/Flu) surface-functionalized (OS/Flu-PEI) to find an optimal combination of the cells and OS-PEIs. Then, we performed *in vitro* cell labeling using OS/Flu-PEI. We discovered that some cells showed barcoded endosomes with a various pattern of fluorescence due to differential endosomal sorting of the particles depending on the surface of the NP. We performed optical seamless imaging to visualize the *in vitro*-labeled cells. The cells showing barcorded endosomes were a novel type of labeled cells. We conducted intraperitoneal administration of *in vitro*-labeled cells with OS-PEI into a mouse that was also injected with thiol-OS containing rhodamine B (thiol-OS/Rho) to label peritoneal cells *in situ*. The *in vitro*-labeled cells including the ones showing barcoded endosomes forming cell aggregates with *in situ*-labeled peritoneal cells were seamlessly observed from macroscopic imaging *in vivo* to microscopic imaging at the single-cell level *ex vivo*. The seamless imaging of *in vitro*-labeled cells using NPs demonstrated high potential for innovative next-generation imaging.

## Results and discussion

### Preparation and characterization of surface-functionalized fluorescent organosilica NPs with various molecular weight PEIs

**Fig. 1:**
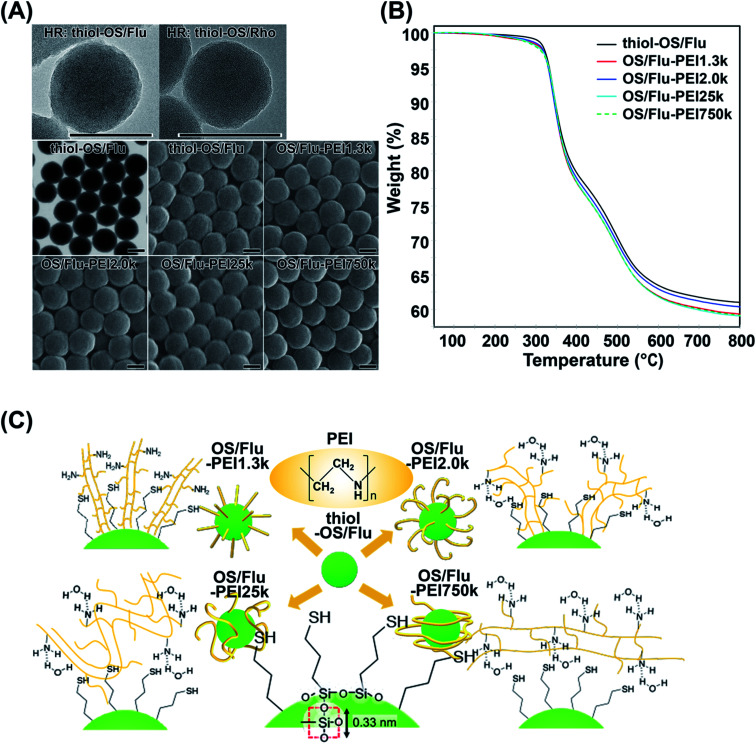
Surface-functionalized fluorescent organosilica nanoparticles with PEIs of various molecular weights. (A) High resolution TEM micrographs of thiol-OS/Flu (HR: thiol-OS/Flu) and thiol-OS/Rho (HR: thiol-OS/Rho), STEM and SEM micrographs of thiol-OS/Flu and OS/Flu-PEIs. Scale bars: 100 nm. (B) Thermal gravimetric analysis of nanoparticles. The data for thiol-OS/Flu (black), OS/Flu-PEI1.3k (red), OS/Flu-PEI2.0k (blue), OS/Flu-PEI25k (cyan), and OS/Flu-PEI750k (green; dotted line) were obtained under flowing nitrogen. (C) Schematic models of thiol-OS/Flu and 4 kinds of OS/Flu-PEIs. A molecular model of the silica network on the surface is shown with the red square of oxygen centered on silicon with a distance of 0.33 nm. The 3-D models of OS/Flu-PEIs show that OS/Flu-PEI1.3k is a brush type, OS/Flu-PEI2.0k is a bent brush type, OS/Flu-PEI25k is a bent lie-down type, and OS/Flu-PEI750k is a coiled type.

Thiol-OS/Flu and thiol-OS/Rho were synthesized according to our previous report.^18^ High resolution TEM showed gentle irregularities on the surface and dot patterns on the inside might indicate Si atoms. STEM and SEM revealed that thiol-OS/Flu had a spherical shape, narrow size distribution, and good dispersion (Fig. 1A). The surface functionalization of thiol-OS/Flu with PEIs was carried out using electrostatic interactions between the negative charge of thiol-OS/Flu and the positive charge of the PEIs. Thiol-OS/Flu was mixed and stirred with solutions containing 4 different MWs of branched PEI, and the PEI bound onto the NP surface. All OS/Flu-PEIs were observed by SEM after osmium coating. The surface of the OS/Flu-PEIs did not show a clear difference from that of thiol-OS/Flu (Fig. 1A). In the DLS analysis, the hydrodynamic particle diameter of thiol-OS/Flu was larger than that measured by STEM (Table 1), as reported previously.^17,19^ The diameters calculated based on SEM and the hydrodynamic diameters of the OS/Flu-PEIs were greater than that of thiol-OS/Flu. The diameters of the 4 OS/Flu-PEIs were almost the same but the diameters of OS/Flu-PEI1.3k and OS/Flu-PEI25k were the largest and the smallest based on SEM and DLS, respectively. The increase in hydrodynamic diameter did not depend on the MW. The 3-D structure of PEIs on the surface of thiol-OS/Flu might affect their hydrodynamic diameters. The coefficients of variation (% PD) and polydispersity index (PDI) of the thiol-OS/Flu diameter measured by both EM and DLS were lower than those of the OS/Flu-PEIs. The % PDs and PDIs of OS/Flu-PEIs, except for OS/Flu-PEI750k, were at least 1.5-fold higher than that of thiol-OS/Flu. Previously, we performed surface functionalization of thiol-OS/Flu with various MWs of polyethylene glycol (PEG).^17^ The coefficients of variation of thiol-OS/Flu with PEG ranged from 0.77 to 1.34%, close to the 1.08% for thiol-OS/Flu.^20^ The 3-D structure of PEI on the surface varies from particle to particle more than that of PEG. The % PDs of thiol-OS/Flu with lower-MW PEIs (OS/Flu-PEI1.3k and OS/Flu-PEI2.0k) (OS/Flu-PEI-L) were higher than those of thiol-OS/Flu with higher-MW PEIs (OS/Flu-PEI25k and OS/Flu-PEI750k) (OS/Flu-PEI-H). These data indicated that the 3-D structure of the PEI of OS/Flu-PEI-L varies more than that of OS/Flu-PEI-H. Thiol-OS/Flu had a negative *ζ* potential due to the thiol and silanol residues on the surface. The OS/Flu-PEIs had a positive *ζ* potential due to the intramolecular amino group of the PEIs on their surface. The *ζ* potentials of the OS/Flu-PEIs were not the same. OS/Flu-PEI1.3k and OS/Flu-PEI25k had similar values, which were higher than that of OS/Flu-PEI750k and lower than that of OS/Flu-PEI2.0k. These differences would be related to the density of the PEIs on the particle surface and their 3-D structure.

**Table 1:** Particle sizes and *ζ* potentials of OS/Flu-PEIs^a^

	Diameter^b^ (nm) (SEM)	Diameter (nm) (DLS)	%PD^c^	PDI	*ζ* potential (mV)
Thiol-OS/Flu	151 ± 6.7	132.1 ± 9.0	16.2 ± 3.8	0.040	−30.6 ± 4.8
OS/Flu-PEI1.3k	159 ± 8.2	185.1 ± 6.3	34.5 ± 4.4	0.086	29.1 ± 2.4
OS/Flu-PEI2.0k	156 ± 8.4	160.5 ± 14.3	26.3 ± 8.1	0.110	32.5 ± 8.1
OS/Flu-PEI25k	154 ± 8.9	144.9 ± 4.0	17.1 ± 8.8	0.067	29.0 ± 4.9
OS/Flu-PEI750k	156 ± 7.6	167.5 ± 8.1	16.4 ± 6.7	0.059	27.1 ± 1.0

a Each value represents the mean ± standard deviation (SD) of 3 replicates.

b The mean diameter and SD of nanoparticles were calculated from more than 40 particles measured by SEM.

c PD (%) was calculated as ((polydispersity *×* 2)/diameter) *×* 100.

To evaluate the PEI densities on the OS/Flu-PEIs, we performed TGA (Fig. 1B and Table 2). The initial weight loss at 200 °C was due to the desorption of water molecules from the surface of the particle. The remaining weights were 60.75%, 59.81%, 60.05%, 59.58, and 59.29% for thiol-OS/Flu, OS/Flu-PEI1.3k, -PEI2.0k, -PEI25k, and -PEI750k, respectively, at 300–800 °C under flowing nitrogen. The estimated PEI weight, number per particle, and density on thiol-OS/Flu are summarized in Table 2. The PEI parts of OS/Flu-PEI1.3k and OS/Flu-PEI25k were similar, and those of OS/Flu-PEI2.0k and OS/Flu-PEI750k were the lowest and the highest, respectively. Previously, thiol-OS/Flu surface-functionalized with methoxy-PEG-maleimide with MWs of 12k and 30k was prepared, and the PEG parts measured by TGA were 1.82% and 4.73%, respectively.^17^ The PEG part of thiol-OS/Flu surface-functionalized with methoxy-PEG-maleimide was higher than the PEI part of OS/Flu-PEI25k. These results indicated that the binding efficacies of the 4 kinds of PEI *via* electrostatic and hydrophobic interactions were not higher than those of PEG obtained *via* the conjugation of thiol and maleimide. The ratio of mercaptopropyl residues to PEI1.3k was estimated to be 17 : 1, the maximum among PEIs. The PEI density on particles and the MW of PEI were inversely correlated except for OS/Flu-PEI2.0k. The PEI density of OS/Flu-PEI2.0k was the lowest, approximately 62%, compared with that of OS/Flu-PEI1.3k, although the difference in MW was not large. These results indicated that PEIs were bound to the surface of thiol-OS in various forms depending on their MW. We estimated and have shown the models of the forms of PEI on thiol-PS/Flu in Fig. 1C.

**Table 2:** Thermal gravimetric analyses of thiol-OS/Flu and the 4 kinds of OS/Flu-PEIs^a^

	Thiol-OS/Flu	OS/Flu-PEI1.3k	OS/Flu-PEI2.0k	OS/Flu-PEI25k	OS/Flu-PEI750k
Weight^a^ at 200 °C (%)	99.69 ± 0.07	99.76 ± 0.20	99.62 ± 0.09	99.64 ± 0.07	99.67 ± 0.11
Weight^a^ at 800 °C (%)	60.75 ± 0.45	59.81 ± 0.47	60.05 ± 0.63	59.58 ± 0.44	59.29 ± 0.16
Organic part^a^ (%)	38.94 ± 0.39	39.95 ± 0.28	39.57 ± 0.56	40.06 ± 0.46	40.38 ± 0.04
PEI part (%)	—	1.01 ± 0.28	0.63 ± 0.56	1.12 ± 0.46	1.44 ± 0.04
PEI weight (g)/particle	—	3.00 × 10^−17^ ± 0.09 × 10^−17^	1.87 × 10^−17^ ± 1.66 × 10^−17^	3.33 × 10^−17^ ± 1.38 × 10^−17^	4.28 × 10^−17^ ± 0.12 × 10^−17^
PEI number/particle		13 890 ± 417	5640 ± 5007	802 ± 332	35 ± 1
PEI density (PEI/nm^2^)		0.583 ± 0.162	0.237 ± 0.211	0.034 ± 0.014	0.001 ± 0.000

a Each value represents the mean ± SD of 3 replicates.

PEIs contain hydrophobic (ethylene groups) and positively charged hydrophilic (amino groups) parts. It was reported that the structure of linear PEI could be changed; it was elongated in fully protonated states (lower pH) but in a coiled form in nonprotonated states (higher pH) in liquid.^21^ Thiol-OS has a negatively charged hydrophilic (thiol in mercaptopropyl residues and silanol residues) part and a hydrophobic (propyl groups) part on the surface. The maximum density of mercaptopropyl residues on the surface was theoretically estimated at 10 residues per nm^2^ because the distance between one side of the square of oxygen centered on silicon was 0.33 nm. Therefore, PEI present on the surface of thiol-OS/Flu formed various structures due to electrostatic and hydrophobic interactions with the residues on thiol-OS. The 3-D structures of PEIs on the particles were not the same as those in liquid. The 3-D structures of PEG on the surface of particles have been well investigated and discussed, with forms described as, for example, brush^22^ and mushroom^23^ types. These forms were determined based on their MW and the surface density of the PEG on the NPs. We estimated and proposed models of the 3-D structures of PEI based on their MWs and the surface densities as well as *ζ* potentials and diameters. The *ζ* potentials of OS/Flu-PEIs indicated an electrostatic interaction between negatively charged residues such as thiol and silanol on the particle surface and the amino residues of PEIs. The diameters of OS/Flu-PEIs could be related to their style on the particle surface such as horizontal and vertical. We proposed that OS/Flu-PEI1.3k was a brush type, OS/Flu-PEI2.0k was a bent brush type, OS/Flu-PEI25k was a bent lie-down type, and OS/Flu-PEI750k was a coiled type as shown in Fig. 1C. OS/Flu-PEI1.3k showed the highest diameter on DLS and on SEM, and the highest PEI density on TGA. PEI1.3k might fit into the gaps between the mercaptopropyl chains and could stand nearly vertically on the surface because of the hydrophobic interactions between the aliphatic groups of both the PEI and mercaptopropyl groups (Fig. 1C, OS/Flu-PEI1.3k). Therefore, we proposed that PEI1.3k on OS/Flu-PEI1.3k had a brush form. OS/Flu-PEI2.0k showed no increase in the hydrodynamic diameter compared with that of OS/Flu-PEI1.3k and the lowest PEI weight/particle. These data indicated that PEI2.0k was not fully upright and extended as linear on the surface. PEI2.0 was bent on the surface, and its bent conformation inhibited the binding of PEI on the surface of particles to each other. The positive value of the *ζ* potential of OS/Flu-PEI2.0k was the highest among the 4 OS/Flu-PEIs. This indicates that negatively charged residues such as thiol and silanol on the particle were masked by electrostatic interactions with the amino residues of PEI 2.0k, including larger proportions of bent conformations than other PEIs. Therefore, we proposed that OS/Flu-PEI2.0k had a bent brush form (Fig. 1C, OS/Flu-PEI2.0k). OS/Flu-PEI25k showed the shortest diameter on DLS and SEM. PEI25k might bind horizontally to the particle surface, but not vertically like PEI1.3k. OS/Flu-PEI25k showed a lower positive value of the *ζ* potential but not a lower PEI weight/particle than OS/Flu-PEI2.0k. Therefore, PEI25k on the surface might be well arranged without great structural inhibition like that of PEI2.0k. And PEI25k exists on the surface in a form with relatively little interaction with the negatively charged residues of the particles. As a possible model, we proposed that PEI25k adopted a bent lie-down form on thiol-OS/Flu (Fig. 1C, OS/Flu-PEI25k). The bent lie-down form of PEI might be caused by hydrophobic interactions between the ethylene groups of PEI and propyl residues of particles on the inner surface rather than thiol residues at the end of mercaptopropyl residues. Therefore, PEI25k bound with the surface closer than other PEIs, and formed a bent lie-down form. OS/Flu-PEI750k showed a diameter similar to that of OS/Flu-PEI-L, which had the highest PEI weight/particle and the lowest *ζ* potential among OS/Flu-PEIs. These results indicated that PEI750k binds also horizontally to the particle surface. But PEI750k did not interact very much with hydrophobic residues or negatively charged residues on thiol-OS/Flu. Because PEI750k had the highest PEI weight/particle and MW, we estimated that PEI750k contained noncontact parts with particles and existed in the form of a coil around the particles, as shown in Fig. 1C (OS/Flu-PEI750k). These various data indicated that the 3-D structures of PEIs on the particle surface were not the same as those in liquid depending on their MW and their interaction type such electrostatic and hydrophobic interactions. “Molecule-surface residue interaction on particle” is also very important to understand and to control the function of the molecules such as polymers on the particle surface. We reported that various kinds of organosilica particles caused exposure not only to the mercaptopropyl residue^24–26^ but also to the epoxycyclohexyl residue.^27^ These particles would contribute to a study on molecule–surface residue interactions on particles. Although there was not enough evidence to determine the PEI structure on the surface of thiol-OS/Flu, we proposed a model of each NP based on the TEM, SEM, DLS, and TGA results. We believe these models to be important because these 4 types of OS/Flu-PEIs exhibited different effects on cells, as described below. Further studies were required to evaluate the surface structure of PEIs on the particles to understand cell–NP interactions in addition to molecule–surface residue interactions on particles. Evaluations of the 3-D structure of PEI on the particle surface using SEM observation, with a possible goal of enhancing the PEIs, are in progress.

#### Aggregation-redispersion phenomenon of OS/Flu-PEIs depending on the PEI concentration

**Fig. 2:**
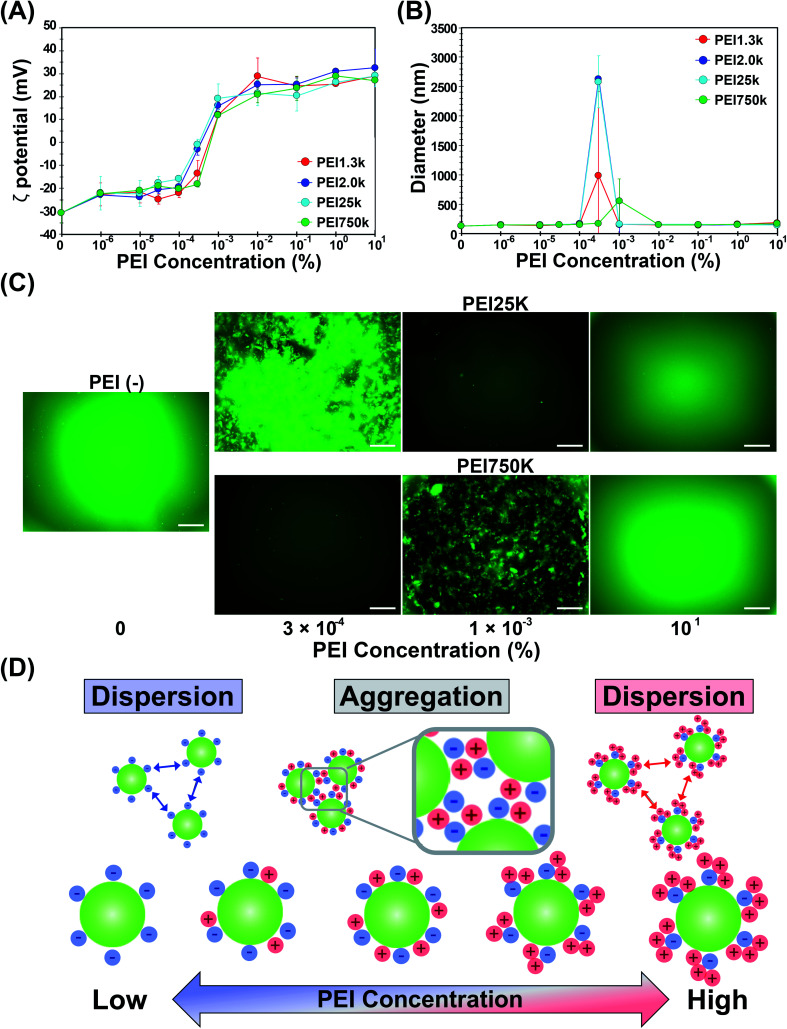
Aggregation-redispersion phenomenon of OS/Flu-PEI. The relationship between the concentrations of the 4 kinds of PEIs (PEI1.3k (red), PEI2.0k (blue), PEI25k (cyan), and PEI750k (green)) and *ζ* potential (A) and the hydrodynamic diameter (B) of OS/Flu-PEIs were analyzed by dynamic light scattering. (C) On microscopic observations, thiol-OS/Flu and OS/Flu-PEI25k prepared from 10% PEI showed good dispersion, but OS/Flu-PEI25k prepared from 3 × 10^−4^% PEI and OS/Flu-PEI750k prepared from 1 × 10^−3^% PEI showed large fluorescent aggregates of NPs. Scale bars: 2 μm. (D) The homogenous and heterogeneous distribution of the negative charge (blue) of the surface of thiol-OS/Flu and the positive charge (red) of PEI are shown as a model.

Because the 4 kinds of OS/Flu-PEIs revealed different physicochemical characteristics and were estimated to have different 3-D structures on the surface depending on the MW of PEI, we investigated the relationship between the PEI concentration and both the *ζ* potential and the hydrodynamic diameter of each OS/Flu-PEI using DLS. As shown in Fig. 2A, the relationship between the PEI concentration and the *ζ* potential of the 4 kinds of OS/Flu-PEIs showed characteristic changes. At lower concentrations from 1 × 10^−6^% to 1 × 10^−4^% PEIs, the increases in *ζ* potential were small. From 3 × 10^−4^% to 1 × 10^−2^% PEIs, the *ζ* potential increased greatly to a positive charge. Above 1 × 10^−2^% PEIs, the changes in the *ζ* potential were small again. PEI25k showed a more efficient increase than the others. OS/Flu-PEI25k showed a slight early increase from 3 × 10^−5^% to 1 × 10^−3^% PEIs. We hypothesized that OS/Flu-PEI25k adopted a bent lie-down form due to hydrophobic interactions on the surface of thiol-OS/Flu. PEI25k might have bound to the surface at a lower concentration of 3 × 10^−5^% PEI under hydrophobic driving. The binding of PEI25k with the particle surface was completed at 3 × 10^−4^% PEI, and the *ζ* potential became stable above 3 × 10^−4^% PEI. Other PEIs also showed subtle differences. PEI750k might have bound to the surface at a concentration of 1 × 10^−3^% PEI and was completed at 1 × 10^−2^% PEI. The binding and the stabilization of PEI750k with the surface of thiol-OS/Flu required a higher concentration than the others. PEI750k reflected changes in interactions with thiol-OS/Flu, such as electrostatic or hydrophobic interactions, due to differences in the 3-D structure of PEIs in liquid. As shown in Fig. 2B, the hydrodynamic diameter showed a transient increase at 3 × 10^−4^% PEI for OS/Flu-PEI1.3k, -PEI 2.0k, and -PEI 25k and at 1 × 10^−3^% PEI for OS/Flu-PEI750k. The hydrodynamic diameters of OS/Flu-PEI2.0k and -PEI25k showed the greatest change, increasing from about 150 nm at 10^−5^% PEI to 2600 nm at 3 × 10^−4^% PEI. The hydrodynamic diameter of OS/Flu-PEI1.3k showed a great inter-experimental difference of increases at 3 × 10^−4^% PEI. Above 1 × 10^−2^% PEI, the hydrodynamic diameters of all OS/Flu-PEIs decreased and remained constant. On fluorescence microscopic observation (Fig. 2C), thiol-OS/Flu, as well as OS/Flu-PEI25k prepared from 10% PEI, showed good dispersion. However, OS/Flu-PEI25k prepared from 3 × 10^−4^% PEI and OS/Flu-PEI750k prepared from 1 × 10^−3^% PEI showed microsized fluorescent aggregates of NPs which could also be observed as microscopic precipitation. These findings indicated that the transient increases in the hydrodynamic diameter were due to NP aggregation, where the dispersed NPs temporarily aggregated at a particular concentration and then redispersed at a higher concentration. These results demonstrated that thiol-OS/Flu treated with various concentrations of PEI showed a clear PEI concentration-dependent aggregation-redispersion phenomenon. As a related phenomenon, the precipitation and redispersion of NPs coated with poly(acrylic acid) (PAA) were reported and applied for the stable dispersion of NPs.^28–30^ As a well-known result, ceria NPs coated with PAA as a short polyelectrolyte brush surrounding the NPs showed macroscopic precipitation at low pH. As the pH increased, precipitated NPs were redispersed as single NPs with an anionic poly(acrylic acid) corona.^28^ This approach was applied to various NPs. In our study, the aggregation-redispersion phenomenon observed for thiol-OS/Flu and PEIs was very simple and provided the possible mechanisms of the aggregation and redispersion of NPs. The aggregation of OS/Flu-PEIs was reversible to dispersion of NPs as they dispersed at higher PEI concentrations. We propose that the aggregation of OS/Flu-PEIs was caused by the heterogeneity of the charge distribution on the particle surface, as shown in Fig. 2D. At a PEI concentration of 3 × 10^−4^%, the *ζ* potentials of OS/Flu treated with PEI1.3k, PEI2.0k, and PEI25k were relatively neutral, −13.5, −2.9, and −1.0 mV, respectively, and showed transient aggregation. At this time, the negative charge of the silanol and thiol groups derived from thiol-OS/Flu and the positive charge of the amino group derived from PEI might cancel their charges on the particle surface and reveal a neutral charge on DLS. In addition to the surface of NPs being neutral in terms of *ζ* potential, we proposed a heterogeneity model of the distribution of negative and positive charges on the particle surface as shown in Fig. 2D. Because both a positive and a negative charge coexisted on the same particle surface, a positively charged region derived from PEIs could interact with a negatively charged region of another particle, and aggregation could occur. On the other hand, at a lower PEI concentration of less than 1 × 10^−4^% or a higher concentration more than 1 × 10^−2^%, a negative or positive charge becomes predominant on one side. At a lower PEI concentration, the negative charged surface due to thiol and silanol residues caused repulsion between particles. At a higher PEI concentration, the positively charged surface covered with PEI on the negative charge of thiol and silanol residues also caused repulsion between particles. Therefore, the NPs are redispersed after aggregation due to electrostatic repulsions from the homogeneous positive charge of PEIs covering the surface of the NPs. The concentration-dependent aggregation-redispersion using PEIs and thiol-OS/Flu could be a simple and good model to discuss the importance of the concentration of molecules for surface functionalization.

#### Evaluation of cell labeling efficacy

**Fig. 3:**
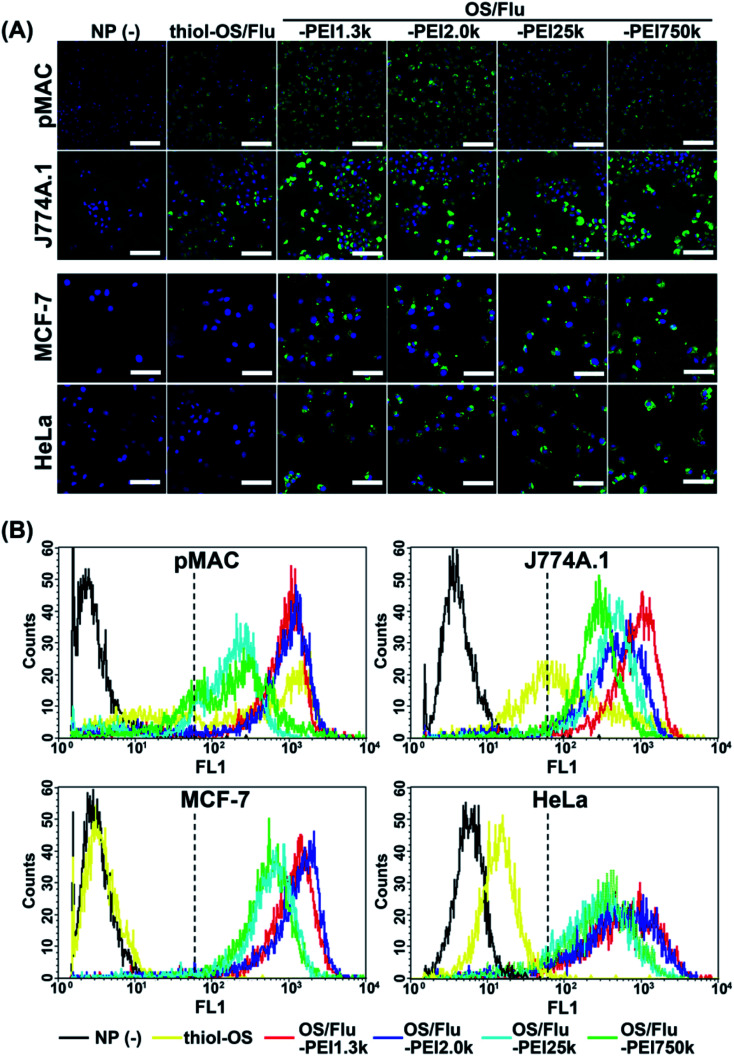
Fluorescence microscopic observation and flow cytometric analysis of cells labeled with OS/Flu-PEIs. (A) The cells were treated with thiol-OS/Flu, and 4 kinds of OS/Flu-PEIs were observed by fluorescence microscopy. Scale bars: 50 μm. (B) The cells were treated with thiol-OS/Flu (yellow), OS/Flu-PEI1.3k (red), OS/Flu-PEI2.0k (blue), OS/Flu-PEI25k (cyan), and OS/Flu-PEI750k (green) or without particles (black) and subjected to FCM. The intensity of the criterion for positive labeling is indicated by the dotted line.

The efficiency of the cell labeling of OS/Flu-PEIs was evaluated using fluorescence microscopic observation and flow cytometry (FCM) analysis (Fig. 3). Four kinds of cells, including cancer cells (human cervical cancer HeLa cells and human breast cancer MCF-7 cells) and macrophages (mouse cell line J774.1 and a primary culture (mouse peritoneal macrophages (pMAC))), were treated with each NP. All OS/Flu-PEIs could label these 4 kinds of cells. In fluorescence microscopy, J774.1 cells and mouse peritoneal macrophages labeled with OS/Flu-PEIs showed higher fluorescence intensity than those labeled with thiol-OS/Flu. Surface functionalization with PEIs of thiol-OS/Flu enhanced cell labeling efficacy compared with the native uptake abilities of macrophages. Two cancer cell lines showed clear fluorescence with all OS/Flu-PEIs but not with all thiol-OS/Flu.

**Fig. 4:**
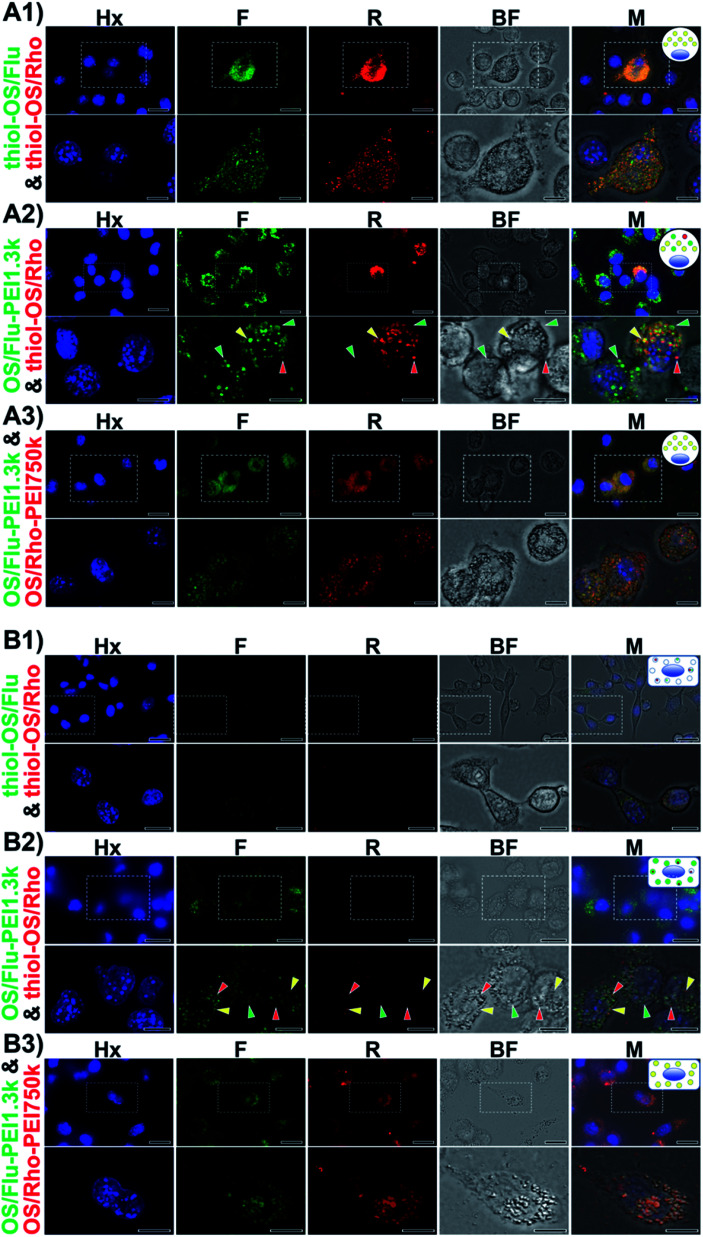
High-resolution fluorescence microscopic observation of dual-particle-treated cells. J774A.1 cells (A) and HeLa cells (B) were treated with thiol-OS/Flu and thiol-OS/Rho (A1 and B1), OS/Flu-PEI1.3k and thiol-OS/Rho (A2 and B2), or OS/Flu-PEI1.3k and OS/Rho-PEI750k (A3 and B3), respectively. Images of the blue fluorescence (Hx) from Hoechst 33342 staining, green from OS/Flu (F), red from thiol-OS/Rho (R), bright field (BF), and merged images (M) are shown. Scale bars: upper panels: 10 μm, lower panels: 20 μm.

Next, we conducted flow cytometry to quantitatively evaluate labeling efficacy (Fig. 3B and Table 3). The cell labeling efficacy was evaluated using the labeling ratio (labeled cells per all cells (%)) and labeling intensity (fluorescence intensity (F.I.)) of labeled cells. The cells with a geometric mean above 62 on FL2 were defined as labeled cells, and labeling intensity was calculated based on the geometric mean of labeled cells. As shown in Fig. 3B, the labeling profiles of each cell using NPs were also different for each cell type and each NP. The labeled cells using thiol-OS/Flu were detected at a high labeling ratio in macrophage cells but rarely in cancer cells, similar to the findings of fluorescence microscopy. However, the HeLa cells treated with thiol-OS/Flu showed a small right shift compared with the cells without NP treatment which will be discussed later with microscopic observation at high magnification (Fig. 4B). The cells except for pMAC revealed a high labeling ratio to OS/Flu-PEIs, above 90% (Table 3). However, the labeling intensity was different for each cell type and each NP. Some cells revealed different labeling efficacies depending on the PEI MW. In pMAC and MCF-7 cells, the labeling intensities between the two particles of OS/Flu-PEI-L were close, and the labeling intensities of OS/Flu-PEI-L were approximately 2-fold higher than those of OS/Flu-PEI-H. In HeLa cells, the labeling intensities of OS/Flu-PEI-L were higher than those of OS/Flu-PEI-H. However, in J774A.1, the labeling intensities of OS/Flu-PEI-L showed a large difference. The labeling intensity of OS/Flu-PEI2.0k was approximately 56.7% of that of OS/Flu-PEI2.0k, which was rather close to that of OS/Flu-PEI-H.

**Table 3:** Flow cytometric analysis of the 4 kinds of cells labeled with thiol-OS/Flu and the 4 kinds of OS/Flu-PEIs^a^

NPs	pMAC		J774A.1		MCF-7		HeLa	
	Population (%)	Level (F.I.)	Population (%)	Level (F.I.)	Population (%)	Level (F.I.)	Population (%)	Level (F.I.)
Thiol-OS/Flu	72.2 ± 9.4	639.1 ± 153.4	61.1 ± 4.8	204.0 ± 30.1	1.7 ± 1.4	157.8 ± 99.7	0.2 ± 0.2	111.3 ± 32.5
OS/Flu-PEI1.3k	84.9 ± 12.0	815.0 ± 125.6	96.4 ± 0.4	815.7 ± 105.4	96.8 ± 1.9	1038.9 ± 248.3	95.3 ± 2.9	589.8 ± 63.9
OS/Flu-PEI2.0k	79.9 ± 12.0	812.3 ± 193.1	95.7 ± 0.9	462.6 ± 36.0	96.7 ± 1.0	1026.3 ± 292.3	93.6 ± 4.9	501.6 ± 171.6
OS/Flu-PEI25k	78.0 ± 7.9	283.3 ± 145.0	96.0 ± 1.2	360.6 ± 88.8	96.3 ± 0.2	588.1 ± 187.7	89.9 ± 2.0	251.9 ± 87.9
OS/Flu-PEI750k	82.8 ± 7.0	394.4 ± 118.9	95.3 ± 1.0	278.8 ± 85.2	86.7 ± 12.8	417.9 ± 185.5	92.1 ± 5.5	365.5 ± 165.1

a Each value represents the mean ± SD of 3 replicates.

The labeling ratio of OS/Flu-PEIs in pMAC was lower than those of other cells. And the increases in the labeling ratio from that of thiol-OS/Flu of pMAC were lower than those of J774A.1 cells. Among OS/Flu-PEIs, their labeling ratio did not show a predominant difference, but the labeling intensity showed a great difference. The labeling intensities of OS/Flu-PEI-L were increased, but not those of OS/Flu-PEI-H, compared with those of thiol-OS/Flu. A lower labeling intensity of OS/Flu-PEIs than thiol-OS/Flu was observed only in pMAC with OS/Flu-PEI-H among all 4 kinds of cells. In J774A.1 cells, the labeling ratios of all OS/Flu-PEIs were above 95% and higher than those of pMAC and HeLa cells. The labeling intensities of OS/Flu-PEIs were higher than those of thiol-OS/Flu and showed an inverse correlation with PEI MW. In MCF-7 cells, the labeling ratio except for OS/Flu-PEI750k and the labeling intensities of all OS/Flu-PEIs were the highest among the 4 kinds of cells. The labeling intensities of OS/Flu-PEIs also showed an inverse correlation with the PEI MW. In HeLa cells, the labeling ratios of all OS/Flu-PEIs were above approximately 90%. However, the labeling intensities of OS/Flu-PEIs were the lowest among all 4 kinds of cells except for that of OS/Flu-PEI750k.

Because FCM analysis showed much lower intensity cells with OS/Flu-PEIs specifically recognized in pMAC, we observed the cells again by fluorescence microscopy. As shown in Fig. S1A,† we found unlabeled or extremely low-labeled cells in pMAC with OS/Flu-PEIs. Additionally, unlabeled or extremely low labeled cells with OS/Flu-PEIs were found in the other 3 kinds of cells. The reason cells could not be labeled with OS/Flu-PEI is that they are dead or have low uptake activity. Interestingly, we found J774A.1 cells that could be labeled with thiol-OS without PEI but not OS/Flu-PEI1.3k, as shown in Fig. S1B.† (This finding was associated with intracellular labeling using dual-particle administration in the experiments described in the next section.) This cell indicated that the cell had a mechanism that could eliminate the interaction with OS/Flu-PEI1.3k. Previously, we reported the existence of PEG-resistant macrophages on stealth imaging *in vitro* using thiol-OS/Flu surface-functionalized with PEG.^17^ PEG-resistant macrophages could resist the stealth function of PEG and take up thiol-OS/Flu surface-functionalized with PEG at the same level as thiol-OS without PEG. We found cells that could be labeled with thiol-OS/Rho but not OS/Flu-PEI1.3k very well, indicating the existence of PEI-resistant macrophages that resist interaction with PEI on NPs. Further characterization and investigation of PEI-resistant macrophages are required to understand the molecular mechanism of resistance to PEI binding, such as electrostatic interactions.

The labeling ratio and intensities of OS/Flu-PEI1.3k were the highest compared with other NPs in all 4 kinds of cells. For OS/Flu-PEI2.0k, the labeling ratio was close to that of OS/Flu-PEI1.3k, and the labeling intensities were also the highest except for J774A.1. The labeling ratio and intensities of OS/Flu-PEI25k were the lowest in pMAC and HeLa cells, and those of OS/Flu-PEI750k were the lowest in J774A.1 and MCF-7 cells. Therefore, these 4 kinds of OS/Flu-PEIs had different labeling efficacies. Considering our proposed model of OS/Flu-PEIs, the brush type of OS/Flu-PEI1.3k or bent brush type of OS/Flu-PEI2.0k had higher cell labeling efficiency, and the bent lie-down type of OS/Flu-PEI25k and coiled type of OS/Flu-PEI750k had lower efficiency. The efficiency of cell labeling was higher if PEI was placed close to perpendicular to the surface of the particle rather than horizontally against pMAC and MCF-7 cells. The vertical placement of PEI with respect to the surface of the particle might have increased the roughness of the particle surface and increased the surface area for binding to cells. On the other hand, the horizontal placement of PEI on the surface made the particle surface relatively smooth and did not increase the surface area very much. The DLS analysis showed no increase in the diameters of OS/Flu-PEI-H compared with those of OS/Flu-PEI-L. For the surface charge of NPs, the *ζ* potential of OS/Flu-PEI25k was higher than that of OS/Flu-PEI1.3k, but the labeling efficacy of OS/Flu-PEI25k for all cells was lower than that of OS/Flu-PEI1.3k. Therefore, surface charge could not be the most important factor for cell labeling using particles. We considered that the 3-D structure of PEI on the particle was one of the most important factors. These PEIs had the same repeating sequence of (C_2_H_4_NH)_*n*_ as the primary structure, interacted with the surface residue, and formed a 3-D structure on the surface of thiol-OS/Flu, but PEIs on the particles showed different cell labeling efficiencies depending on their MWs. Therefore, we propose that these experiments using OS/Flu-PEIs could be an important model to describe cell-NP interactions depending on the 3-D structures of surface molecules. As problems, methods to evaluate the 3-D structures of surface molecules on the particle were limited, and this limitation made it difficult to make a definitive discussion. The development of additional methods to evaluate the 3-D structures of molecules on NPs and further investigation to analyze cell–nanoparticle interactions would enable an understanding of the mechanism of their binding to cells, cell responses, and subsequently the control of cell functions.

#### Characterization of intracellular labeling using dual-particle administration

OS/Flu-PEIs could label macrophages and cancer cells with good efficacy. Next, we investigated the characteristics of OS/Flu-PEI labeling at the intracellular level by high-resolution fluorescence microscopy of J774A.1 and HeLa cells. To evaluate the effect of PEI on the NP surface, we applied dual-particle administration. Dual-particle administration enabled us to directly observe the difference in the NP function at the single-cell level.^17,31^ In this study, we conducted simultaneous dual-particle administration using thiol-OS/Flu, thiol-OS/Rho, OS/Flu-PEI1.3k, and OS/Rho-PEI750k to compare the changes in intracellular labeling due to the difference in the surface structure of each NP. The fluorescence spectrum of thiol-OS/Flu and thiol-OS/Rho showed distinguished fluorescence peaks under excitation at 488 nm and 578 nm, respectively (Fig. S2A†). And the photostabilities of both particles were stable under the culture condition (Fig. S2B†). Therefore, the fluorescence of the two particles could be differentiated. The cells were simultaneously administered a dual-particle set of thiol-OS/Flu and thiol-OS/Rho, thiol-OS/Rho and OS/Flu-PEI1.3k, or OS/Flu-PEI1.3k and OS/Rho-PEI750k and were incubated. The incubated cells were subjected to optical sectioning observation by structured illumination microscopy. Both kinds of cells exhibited various characteristics of fluorescence and morphology. Some selected representative findings are shown in Fig. 4. J774A.1 cells were administered with thiol-OS/Flu and thiol-OS/Rho and some cells showed both red and green fluorescence. As shown in Fig. 4A1, one cell showed high fluorescence in both images and intracellular small dots in the bright field image. Cells without clear fluorescence surrounding one cell with fluorescence were observed. In the cells without clear fluorescence, the number of intracellular small dots in the bright field image was relatively small. As shown in lower images with higher magnification, one cell with both fluorescence types revealed many small endosomes showing both red and green fluorescence in the merged image. These small fluorescent endosomes were almost at the same position as intracellular small dots in the bright field image. Additionally, some spots showing red and/or green fluorescence were detected in the surrounding cells. Some of these spots were at the same position as intracellular small dots in the bright field image. These findings indicated that thiol-OS/Flu and thiol-OS/Rho were taken up by the cell and localized in almost the same endosomes. The cells administered with thiol-OS/Rho and OS/Flu-PEI1.3k revealed fluorescent and morphological changes in their endosomes (Fig. 4A2). Some cells showed both red and green fluorescence among many cells, showing only green fluorescence. The cells showing both red and green fluorescence revealed variations in the fluorescence patterns of endosomes. In the bright field image, most cells showed many larger intracellular dots than cells administered with thiol-OS/Rho and thiol-OS/Flu. These findings indicated that OS/Flu-PEI1.3k was taken up by most J774A.1 cells. Some cells showed both red and green fluorescence uptake of both OS/Flu-PEI1.3k and thiol-OS/Rho. In one cell showing both red and green fluorescence in the image with higher magnification, some endosomes showed both red and green fluorescence, but others predominantly showed only red or green fluorescence. These findings indicated that some endosomes predominantly contained thiol-OS/Rho or OS/Flu-PEI1.3k (Movies S1 and S2†). Barcoded endosomes with various fluorescence patterns were identified. Using dual administration of thiol-OS/Rho and OS/Flu-PEI1.3k, endosome barcoded J774A.1 cells could be prepared. Also, a PEI resistant J774A.1 cell showing red fluorescence but not green one was identified clearly (Fig. S2B†). This cell could take up thiol-OS/Rho very much, but not OS/Flu-PEI1.3k, and indicated specific mechanisms to avoid the uptake of OS/Flu-PEI1.3k. The cells administered with OS/Flu-PEI1.3k and OS/Rho-PEI750k showed almost all fluorescence (Fig. 4A3). The intensity of each fluorescence appeared to vary somewhat from cell to cell or from endosome to endosome. These findings indicated that OS/Flu-PEI1.3k and OS/Rho-PEI750k were taken up by most cells and localized in almost the same endosomes depending on the uptake activity of each cell.

HeLa cells administered with thiol-OS/Flu and thiol-OS/Rho did not show fluorescence in the fluorescence images, or clear intracellular small dots in the bright field image at lower magnification (Fig. 4B1). In the images with higher magnification, the cells showed both small red and green fluorescent spots. As shown in Fig. 3B and described above, HeLa cells treated with thiol-OS/Flu showed a small shift in FCM, and this small shift was confirmed as a small amount of thiol-OS uptake in or binding with the cells. The cells administered with OS/Flu-PEI1.3k and thiol-OS/Rho showed green fluorescence in the fluorescence images and many intracellular small dots in the bright field image at lower magnification (Fig. 4B2). In the images with higher magnification, the cells showed smaller red fluorescence spots in addition to many green fluorescence spots. The number of red fluorescent dots was smaller than that of green spots. Green fluorescent spots were at the same positions as intracellular small dots in the bright field image, indicating the endosomal uptake of OS/Flu-PEI1.3k, but some red fluorescence dots were not (Movies S3 and S4†). The HeLa cells administered OS/Flu-PEI1.3k and OS/Rho-PEI750k showed both fluorescence in the fluorescence images and many intracellular larger dots in the bright field image (Fig. 4B3). The intensities of both fluorescence varied slightly from cell to cell. However, the intensities of red and green fluorescence was similar for each cell, and the intensity of each fluorescence of the endosome was almost the same. These findings indicated that OS/Flu-PEI1.3k and OS/Rho-PEI750k were taken up by most cells and localized in almost the same endosomes at approximately the same amount.

Characterization of intracellular labeling using dual-particle administration demonstrated some differences in the endosomes, such as their fluorescent patterns and sizes depending on the types of NPs in labeled cells. J774A.1 cells and HeLa cells administered with thiol-OS/Flu and thiol-OS/Rho, and OS/Flu-PEI1.3k and OS/Rho-PEI750k showed similar endosomal localization. In contrast, the administration of thiol-OS/Rho and OS/Flu-PEI1.3k showed variations in red, green, and both fluorescence in endosomes in the cells as barcoded endosomes. These endosomal barcoded cells were the novel type of fluorescent labeled cells. Although it was possible to label intracellular organelles with various fluorescent dyes and fluorescent proteins, no labeling method had been reported to prepare labeled cells that showed multiple different fluorescence types in one type of organelle such as the endosome. Fluorescence labeling to prepare cells showing barcoded organelles using multifunctional NPs would provide new potential for cell labeling and is expected to develop innovative imaging. Previously, we reported that some peritoneal macrophages could take up thiol-OS/Flu surface-functionalized with PEG, and thiol-OS/Rho also showed variations in red and green fluorescence in their endosomes.^17^ These results indicated that cells could recognize the differences in surface structures, such as PEGs and PEIs, of NPs and endocytose them. Additionally, some endosomes preferentially contained particular NPs depending on the surface structure of NPs, using unknown sorting systems to transfer NPs into the inside of each endosome. This intraendosomal sorting might be associated with the expression of cellular functions after endocytosis. Recently, endocytosis has become a more attractive and important biological phenomenon. Endocytosis intimately associates many important biological processes, such as pathogen entry, antigen presentation, growth and differentiation, and intracellular drug delivery.^32–37^ A more detailed understanding of endocytosis would contribute to the development of not only a more efficient therapeutic nanoparticle but also further medical applications such as antiviral treatment against viruses, including SARS-CoV-2.^38–40^ To understand endocytosis, pathways, the mechanism, the morphology, implicated proteins, and the influence of NP physical properties such as the size and charge have been investigated in great detail and are still being actively investigated. However, intraendosomal sorting is less well known. Intraendosomal sorting could be expected to play important roles, such as an enhancement of the signaling response to molecules in the endosome and improvement of the efficiency of antigen presentation. To clarify these hypotheses, ultraprecise and comprehensive morphological studies of cellular endocytosis using higher resolution and multimodal approaches on a nanoscale, such as correlative light and electron microscopy (CLEM) with 3-D reconstruction,^41–43^ are required as important approaches and are under investigation.

#### Influence on the cell activity of OS/Flu-PEIs

**Fig. 5:**
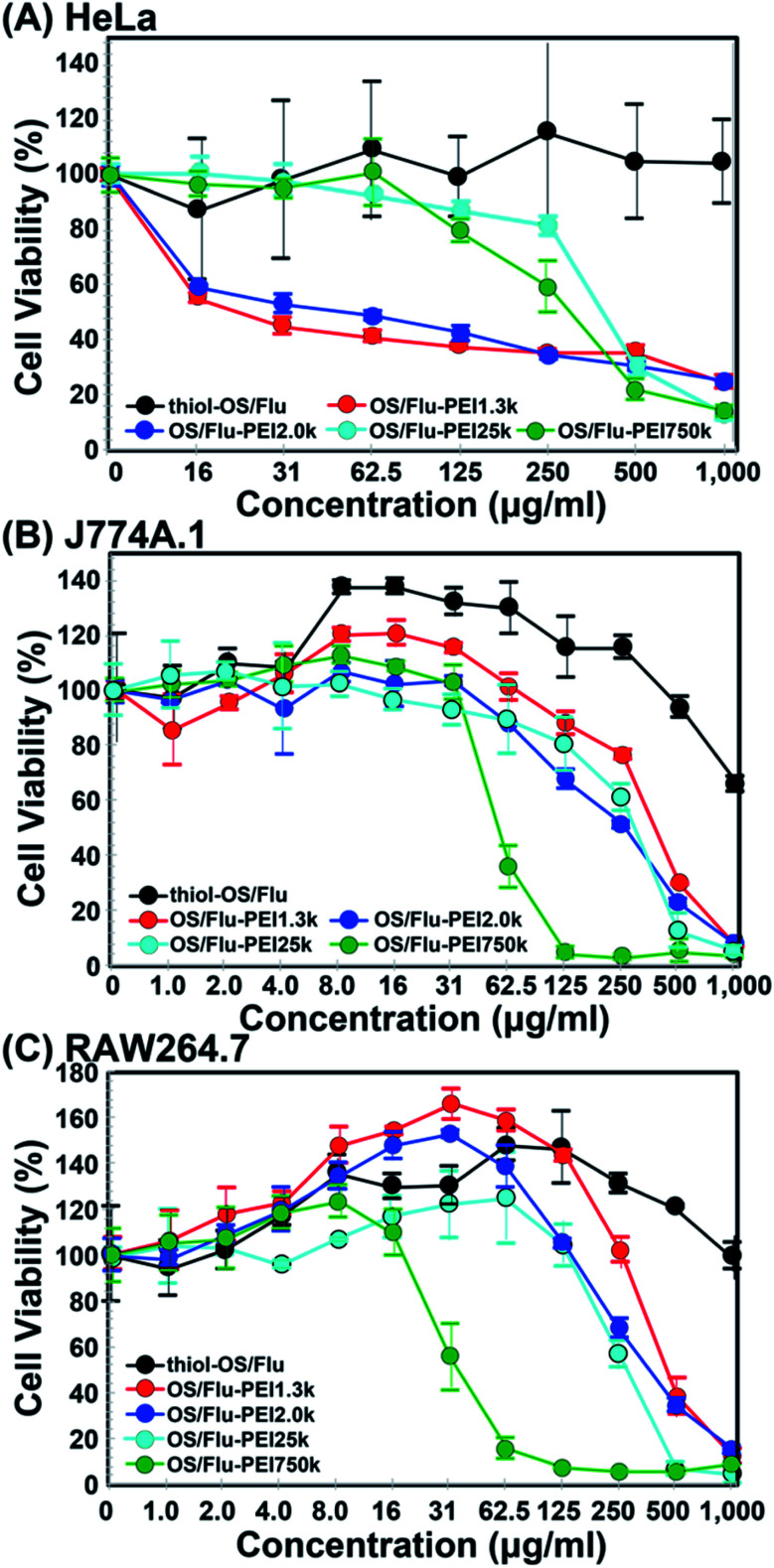
Cell activities of HeLa (A), J774A.1 (B), and RAW 264.7 (C) cells treated with various concentrations of thiol-OS/Flu (black), OS/Flu-PEI1.3k (red), OS/Flu-PEI2.0k (blue), OS/Flu-PEI25k (cyan) and OS/Flu-PEI750k (green) as determined *via* WST-1 assay. Each value represents the mean ± SD of 3 replicates.

We performed a cell activity assay examining the viability of 3 kinds of cultured cells (HeLa, J774A.1, and RAW264.7) in the presence of OS/Flu-PEIs. As shown in Fig. 5A, thiol-OS/Flu did not exhibit readily detectable cytotoxicity in HeLa cells even when the NP concentration was up to 1000 μg mL^−1^. For the macrophage cell line, thiol-OS/Flu showed low cytotoxicity against J774A.1 cells at 1000 μg mL^−1^ but did not show any cytotoxicity against RAW 264.7 cells even at 1000 μg mL^−1^. Previously, we reported that thiol-OS containing a near-infrared fluorescent dye, IR820, also did not show detectable cytotoxicity in 4 kinds of cultured cells (HeLa, human breast cell line MCF-7, J774A.1, and mouse mammary tumor cell line 4T1) up to 100 μg mL^−1^.^44^ In contrast, OS/Flu-PEIs showed concentration-dependent cytotoxicities in these 3 kinds of cells. Therefore, the cytotoxicities induced by OS/Flu-PEIs were derived from the PEIs on the surface of the particles. The LC_50_ (50% lethal concentration) values (concentrations that inhibited cell viability by 50%) of OS/Flu-PEI1.3k, -PEI2.0k, -PEI25k, and PEI750k against HeLa cells were 46.4, 44.5, 358.0, and 286.6 μg mL^−1^. OS/Flu-PEI-L showed higher cytotoxicity than OS/Flu-PEI-H. OS/Flu-PEI-L could label HeLa cells more than OS/Flu-PEI-H at a concentration of 10 μg mL^−1^, as shown in Fig. 5A and B. Therefore, the labeling efficacy of the OS/Flu-PEIs was related to the cytotoxicity in HeLa cells. Among OS/Flu-PEI-L, OS/Flu-PEI1.3k and -PEI2.0k showed similar toxicity, even though the PEI weight/particles of OS/Flu-PEI1.3k was 1.6 times higher than that of OS/Flu-PEI2.0k. These results indicated that toxicity was not dependent on PEI weight/particles. Murine macrophage cell line J774A.1 and RAW 264.7 cells revealed different relations with the PEI MW of OS/Flu-PEIs in cytotoxicity as compared with HeLa cells. The cytotoxicities of OS/Flu-PEIs against macrophage cell lines were PEI MW- and NP concentration-dependent. The LC_50_ values of J774A.1 and RAW 264.7 cells against OS/Flu-PEI1.3k were 537.9 and 456.1, those against OS/Flu-PEI2.0k were 233.5 and 369.3, -PEI25k were 241.6 and 275.6, and -PEI750k were 56.3 and 38.3 μg mL^−1^, respectively. OS/Flu-PEI750k showed the highest cytotoxicity against J774A.1 and RAW 264.7 cells. The OS/Flu-PEI750k had the highest PEI weight/particle, but the labeling efficacy of OS/Flu-PEI750k was the lowest among these 4 OS/Flu-PEIs. OS/Flu-PEI-L showed higher labeling efficacy but not higher cytotoxicity than OS/Flu-PEI-H. Also, the cytotoxicity of OS/Flu-PEI 1.3k was the lowest, even though OS/Flu-PEI1.3k could label J774A.1 cells the highest among OS/Flu-PEIs. Therefore, cytotoxicity was not related to labeling efficacy in J774A.1 and RAW 264.7 cells. Compared with the cytotoxicities of OS/Flu-PEI-L, the cytotoxicity of OS/Flu-PEI 25k was lower than that of OS/Flu-PEI 2.0k in J774A.1 cells and was higher in RAW 264.7 cells. Therefore, the cytotoxicities of the 4 kinds of OS/Flu-PEIs were different for each MW and each cell. OS/Flu-PEI750k had about 10 times higher cytotoxicity than those of OS/Flu-PEI1.3k against J774A.1 and RAW 264.7 cells, even though it has the lowest labeling efficacy. The PEI weight/particle of OS/Flu-PEI750k was just 1.4 times higher than that of OS/Flu-PEI1.3k. We proposed that the higher cytotoxicity of OS/Flu-PEI750k was related to the PEI structure on the surface, such as the coiled type. Further studies including the mechanism of cytotoxicity are required and are under investigation to understand the difference in the cell type- and PEI MW-dependent cytotoxicity of OS/Flu-PEIs.

PEI is the gold standard transfection material for nucleic acid delivery into cells, and the cytotoxicities of PEI molecules and PEI-surface functionalized NPs have been well investigated to improve the efficacy of transfection.^45–48^ It was reported that the cytotoxicity of a low-MW PEI molecule (11k) (LD_50_: approximately 100 μg mL^−1^) was lower than that of high-MW PEI (1616k) (LD_50_: above 35 μg mL^−1^) in human endothelial cells, ECV304 cells and the mouse fibroblast cell line L929.^49^ These results were similar to those of J774A.1 and RAW 264.7 cells but not HeLa cells against OS/Flu-PEIs. Additionally, the LD_50_ values of branched PEI25k for HeLa and human lung carcinoma cell lines and for A549 cells derived from the MTT assay were reported to be 9.93 μg mL^−1^ (ref. 50) and 32.4 μg mL^−1^,^51^ respectively. The LC_50_ values of the amount of PEI converted from OS/Flu-PEI for J774A.1 and RAW 264.7 cells against OS/Flu-PEI1.3k were 5.43 and 4.61, -PEI2.0k were 1.47 and 2.33, -PEI25k were 2.71 and 3.09 and -PEI750k were 0.81 and 0.55 μg mL^−1^ based on the TGA results (Table 2), respectively. The PEI LC_50_ values of OS/Flu-PEI1.3k, -PEI2.0k, -PEI25k, and -PEI750k against HeLa cells were 0.47, 0.28, 4.01, and 4.13 μg mL^−1^, respectively. All LD_50_ values of OS/Flu-PEIs were lower than those of PEI molecules as described above, and the LD_50_ of OS/Flu-PEI25k in HeLa cells was approximately two-fold lower than that of the PEI25k molecule in HeLa cells, indicating that the cytotoxicity of PEI on the surface of OS/Flu-PEI was enhanced slightly. The cytotoxicities of PEI surfaces functionalized with various kinds of NPs were also reported. Surface-functionalized mesoporous silica NPs coated with PEI 0.6, 1.2, 1.8, 10, and 25 kD were evaluated using the MTS assay in the human pancreatic cancer cell lines PANC-1 and BxPC3, RAW 264.7, and bronchial epithelial cell line BEAS-2B.^52^ The LD_50_ values of these cells ranged from approximately 10 μg mL^−1^ to 40 μg mL^−1^. The LD_50_ values of iron oxide NPs coated with PEI25k in RAW 264.7 cells and a human ovary adenocarcinoma cell line, SKOV-3 cells, were between 6.25 and 12.5 μg mL^−1^ and between 3.125 and 6.25 μg mL^−1^, respectively^53^ Because the lowest LD_50_ of OS/Flu-PEIs was approximately 50 μg mL^−1^, OS/Flu-PEIs had lower cytotoxicity than other NPs coated with PEIs. Furthermore, the LD_50_ values of OS/Flu-PEI25k were higher than 240 μg mL^−1^, and the cytotoxicity of OS/Flu-PEI25k was predominantly lower than those of mesoporous silica NPs and iron oxide NPs coated with PEI25k. Because the cell type and particle size were also different, the comparison was not simple, but OS/Flu-PEI was less toxic. Studies of the mechanism of PEI-associated cytotoxicity revealed destabilization of the plasma membrane^54–57^ and the contributions of intracellular organelles such as lysosomes and mitochondria.^58–60^ These mechanisms are also related to the concentration, MW, and structure of PEI. The OS/Flu-PEIs showed concentration-, MW-, and structure-dependent cytotoxicity and contributed to the understanding of the mechanism and then the reduction of the cytotoxicity of PEI using their imaging properties.

**Fig. 6:**
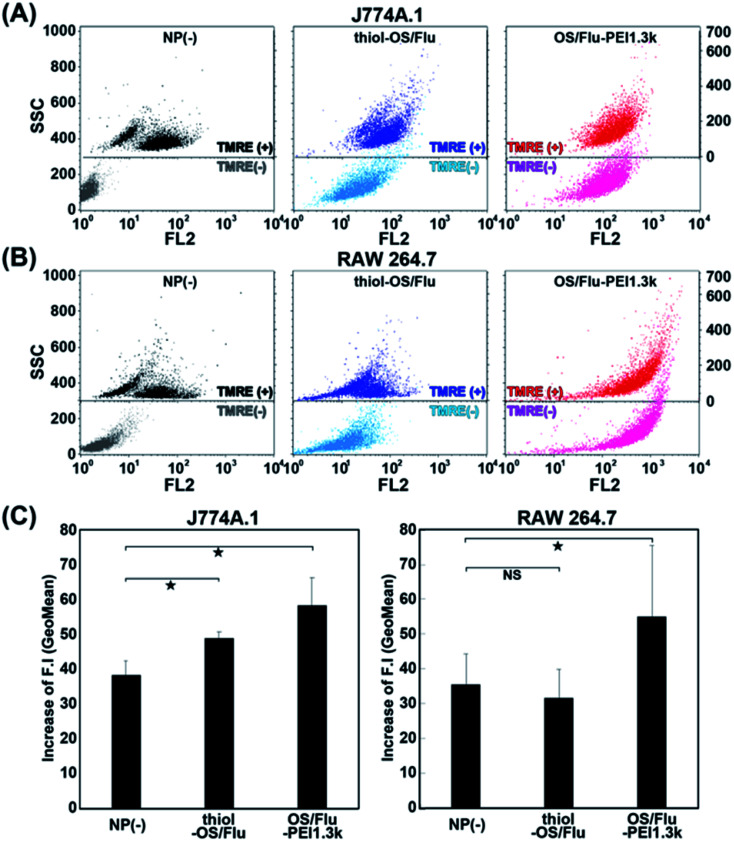
Flow cytometric analysis of J774A.1 (A) and RAW 264.7 (B) cells treated with no nanoparticles, thiol-OS/Flu, and OS/Flu-PEI1.3k and stained with TMRE. The increases in fluorescence intensity (F.I.) were calculated by subtracting the fluorescence intensity (GeoMean) of the cells treated with NPs and stained with TMRE from the control cells treated with NPs but not stained with TMRE. The SSC values of the cells stained without and with TMRE are presented on the right and left sides, respectively. (C) The F.I.s of non-nanoparticles, thiol-OS/Flu, and OS/Flu-PEI1.3k were graphed. Each value represents the mean ± SD of 3 replicates.

Notably, J774A.1 and RAW 264.7 cells showed increases in cell activity (above 120%) depending on NP concentrations and the PEI MW on the particles. Higher increases in the cell activity of J774A.1 cells treated with thiol-OS/Flu from 8.0 to 62.5 μg mL^−1^ and OS/Flu-PEI1.3k from 8.0 to 16.0 μg mL^−1^ were observed. Increases in the cell activity of RAW 264.7 cells treated with OS/Flu-PEI1.3k from 4.0 to 125 μg mL^−1^, thiol-OS/Flu from 4.0 to 500 μg mL^−1^, and OS/Flu-PEI2.0k from 4.0 to 125 μg mL^−1^ were also observed. OS/Flu-PEI25k and -PEI750k showed increases in the cell activity of RAW 264.7 cells at just 62.5 and 8.0 μg mL^−1^, respectively. The increases in the cell activity of RAW 264.7 cells showed for a higher number of kinds of OS/Flu-PEIs and a wider range of NP concentrations than those of J774A.1 cells. To investigate their increases in cell activity, we counted the numbers of J774A.1 and RAW 264.7 cells incubated with 10 μg mL^−1^ thiol-OS/Flu and OS/Flu-PEI1.3k for 1 day. However, the cell number of both cell lines did not increase compared with the control without NP treatment (data not shown). The cell activity was evaluated using WST-1 assay. The WST-1 reagent is cleaved by succinate-tetrazolium reductase in the cellular mitochondria and forms a formazan that can be quantified using light absorbance. Therefore, the results of these experiments indicated alterations in mitochondrial activity, such as the increased enzymatic activity of mitochondrial succinate-tetrazolium reductase. To evaluate mitochondrial activity, we performed staining of the cells using tetramethylrhodamine methyl ester (TMRE). TMRE is a positively charged cellular permeant fluorescent dye that accumulates in the metabolically active mitochondria. The cells incubated with 10 μg mL^−1^ thiol-OS/Flu and OS/Flu-PEI1.3k for 1 day were stained with TMRE, and flow cytometry was applied. As shown in Fig. 6A and B, the flow cytometry profile of the control cells without NP treatment showed two groups with high and low fluorescence intensities. The profiles of the cells treated with NPs and stained with TMRE revealed higher fluorescence intensities than controls treated with TMRE. There was a decrease in the distance between the low and high fluorescence intensity groups in the cells treated with thiol-OS/Flu and an alteration to one group from the two in the cells treated with OS/Flu-PEI1.3k. Because the fluorescence of thiol-OS/Flu was also detected on FL2, the fluorescence intensity (Geo Mean) of the cells treated with NPs and stained with TMRE was subtracted from that of the control cells treated with NPs but not stained with TMRE. The value of the subtracted difference showed an increase in the fluorescence intensity derived from TMRE. As shown in Fig. 6C, significant increases in the TMRE fluorescence intensities of J774A.1 cells treated with thiol-OS/Flu and OS/Flu-PEI1.3k and RAW 264.7 cell treated with OS/Flu-PEI1.3k were observed. Therefore, concentration-dependent and PEI MW-dependent increases in cell activity were derived from the increase in mitochondrial activity. Several studies have reported mitochondrial dysfunction and subsequent cell death^61–68^ induced by various kinds of NPs. As the mechanism of mitochondrial dysfunction, reactive oxygen species triggered by NPs have been well investigated. However, the increase in mitochondrial activity in macrophage cell lines induced by NPs has not been reported. The increase in mitochondrial activity might be associated with the functional activation of macrophages. It was reported that PEI molecules^69^ and PEI-surface functionalized iron oxide NPs^70^ activated macrophages *via* Toll-like receptor 4. Additionally, macrophage activation by functionalized silica NPs was reported. The biocompatible ultrasmall fluorescent core–shell silica NPs (C′ dots) revealed a macrophage-initiated, pseudopathogenic response in the tumor microenvironment.^71^ Both radiolabeled [^225^Ac] alpha MSH-PEG-Cy5-C and unlabeled aMSH-PEG-Cy5- C′ dots upregulated the fraction of M1 macrophages and other immune cells. We reported an accumulation of *in situ*-labeled macrophages with thiol-OS containing IR-820, and the tumor size was observed to be smaller, although was revealed using long-term near-infrared fluorescence *in vivo* imaging of a mouse with a subcutaneous xenograft tumor.^17^ Further investigations are required to understand the activation of macrophages *via* thiol-OS and OS-PEIs and its mechanism. The control of the immune system using NPs is very attractive and important. The NP concentration and PEI MW-dependent macrophage activation using OS/Flu-PEIs would be expected to have some impact on immunotherapy.

#### *In vivo* imaging of *in vitro*-labeled cells combined with *in situ*-labeled cells

**Fig. 7:**
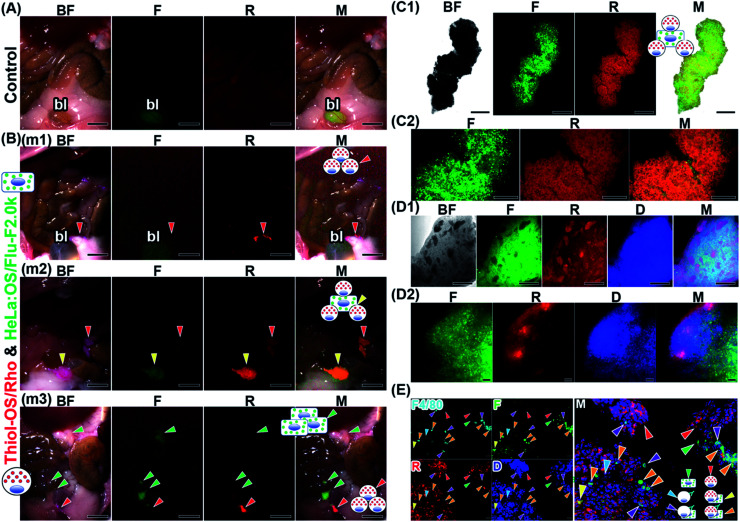
*In vivo* imaging of *in vitro*-labeled cells in the mouse peritoneal cavity. The cultured HeLa cells were labeled with OS/Flu-PEI-2.0k, showing green fluorescence *in vitro*. Thirty minutes before the injection of *in vitro*-labeled HeLa cells, we injected thiol-OS/Rho, which showed intraperitoneal red fluorescence. After 3 h, the peritoneal cavities of the mice were observed. The bright field (BF), green from thiol-OS/Flu (F), red from thiol-OS/Rho (R), bright field (BF), blue from DAPI staining (D), and merged (M) images are shown. (A) A peritoneal cavity of a mouse without any injection was observed on a fluorescence multizoom microscope. Green autofluorescence from the bladder (bl) was observed. Scale bars: 5 mm. (B) The mice injected with thiol-OS/Rho and *in vitro*-labeled cells showed objects with just red fluorescence (red arrowhead) in the mice (m1, m2, and m3), ones with both green and red fluorescence (yellow arrowhead) in a mouse (m2), and ones with just green fluorescence (green arrowhead) in a mouse (m3). Scale bars: 5 mm. (C) An object with both green and red fluorescence obtained from the peritoneal cavity was observed by fluorescence microscopy. (C1) At low magnification, the object showed green and red fluorescence with different patterns. The merged image showed yellow and green fluorescence. Scale bars: 500 μm. (C2) At higher magnification, cells showing red and green fluorescence were observed. The merged image shows the colocalization of both fluorescence in the object and cells with green fluorescence alone. Scale bars: 200 μm. (D) We performed tissue clearing of an object to observe its interior. (D1) The object showed black spots with various shapes and sizes inside (BF). On fluorescence observation, the black spots showed red fluorescence, and the other parts predominantly showed green fluorescence. The merged image separated localization of green and red fluorescence. Scale bars: 500 μm. (D2) At higher magnification, diffuse green fluorescence was observed widely in the object. Red fluorescence was observed linearly along the surface and as small spots near the surface. The merged image showed that the surface predominantly had layered red fluorescence. Small red fluorescent spots also contained green fluorescent cells. Scale bars: 50 μm. (E) Fluorescence immunohistochemistry against a section of a peritoneal object using anti-F4/80 antibody. Some positive cells stained with anti-F4/80 antibody were observed (F4/80), and a cell showed only fluorescence from anti-F4/80 antibody (cyan arrowheads). A larger number of red fluorescent cells (red arrowheads) were observed than green cells (green arrowheads). Additionally, cells showing various fluorescence patterns, such as 3 kinds of fluorescence, including red, green, and fluorescence from anti-F4/80 antibody (orange arrowheads), and two kinds of fluorescence, including fluorescence from anti-F4/80 antibody and green fluorescence (purple arrowheads), were observed. Cells showing both red and green fluorescence (yellow arrowhead) were also observed, indicating F4/80-negative macrophages or other types of cells that could take up thiol-OS/Rho-associated HeLa cells. Scale bars: 20 μm.

We conducted *in vitro* cell labeling and subjected the labeled cells to *in vivo* imaging. Thiol-OS/Flu alone can label macrophage cell lines due to their uptake ability. But the labeling efficacy of thiol-OS/Flu alone was lower than those of OS/Flu-PEIs as shown in Fig. 3 and Table 3. OS/Flu-PEI2.0k was used for cell labeling because of higher labeling efficacy than OS/Flu-PEI-H and lower toxicity than OS/Flu-PEI1.3k at the labeling concentration. We labeled HeLa cells using OS/Flu-PEI2.0k and J774 A.1 cells using OS/Flu-PEI2.0k and thiol-OS/Rho. Then, the labeled cells were intraperitoneally administered to a mouse. The intraperitoneal administration of HeLa cells was examined as an experiment of peritoneal metastatic cancer cell model. After 3 h, we observed the peritoneal cavity, but we could not find labeled cells with good reproducibility (data not shown). Next, we conducted *in vivo* imaging of *in vitro*-labeled HeLa cells with *in situ* labeling of peritoneal cells. Previously, we reported that mouse peritoneal cells could take up thiol-OS *in vitro* very well.^16,17^ Thirty minutes before injection *of in vitro*-labeled cells, we injected thiol-OS/Rho, which showed intraperitoneal red fluorescence, to label the phagocytic peritoneal cells. After 3 h, some objects showing fluorescence in the peritoneal cavity were observed (Fig. 7B). The distribution and fluorescence pattern of the objects differed for each mouse. The mouse shown in Fig. 7B (m1) revealed some objects with red fluorescence, in addition to green autofluorescence from a bladder that was also observed in the control mouse (Fig. 7A). Another mouse (Fig. 7B (m2)) revealed a large object showing both red and green fluorescence and a second object that could detect only red fluorescence. A third mouse (Fig. 7B (m3)) showed 4 objects with only green fluorescence and some with only red fluorescence. The objects with red fluorescence were also observed as a colored red object on a bright field. Next, we performed *ex vivo* fluorescence imaging of the objects showing both green and red fluorescence obtained from the mouse peritoneal cavity. We observed fluorescence microscopy at low magnification (Fig. 7C1). In the bright field, the contour of the object and the difference in light transmittance depending on the site were observed. The object showed both green and red fluorescence with different patterns. The distribution of green fluorescence was scattered. In contrast, the distribution of red fluorescence was diffuse and showed some difference from that of green fluorescence. The merged image with fluorescence and the bright field showed yellow fluorescence and green fluorescence. The yellow fluorescence indicated close localization of labeled HeLa cells and labeled peritoneal cells. At higher magnification (Fig. 7C2), both labeled cells could be observed more clearly. The labeled HeLa cells with green fluorescence located close to labeled peritoneal cells with red fluorescence were well observed. In addition, areas showing only green fluorescence derived from the labeled HeLa cells could be observed. These areas were composed of nonlabeled peritoneal cells as well as labeled HeLa cells. The objects showing both fluorescence were cell aggregates composed of labeled HeLa cells and labeled and nonlabeled peritoneal cells.

We treated another cell aggregate object from the peritoneal cavity with ScaleCubic for tissue clearing to observe its interior region. As shown in Fig. 7D1, the cell aggregate showed black spots of various shapes and sizes inside it, on brightfield observations. On fluorescence observation, the black spots showed red fluorescence, and the other parts surrounding them showed mainly green fluorescence. The surface layer of the cell aggregate showed red fluorescence. The merged image shows magenta fluorescence derived from both DAPI staining of the nuclei and red fluorescence of labeled peritoneal cells and cyan fluorescence derived from both DAPI staining of the nuclei and green fluorescence of labeled HeLa cells. These magenta and cyan fluorescence showed separate positions within the cell aggregate at low magnification. At higher magnification (Fig. 7D2 and Movie S5†), diffuse green fluorescence was observed widely in the cell aggregates. Two kinds of red fluorescence distributions were observed: a linear distribution along the surface and 3 small spots near the surface. The cell shapes of green fluorescence were relatively round, while the shapes of red fluorescence looked elongated and fibrous. The blue fluorescence was observed to be higher in density near the surface and lower toward the inside, indicating the difference in cell density. The merged image showed that the surface showed layered red fluorescence dominantly. The red fluorescent spots near the surface were also surrounded by green fluorescent cells. The area of the small spots also contained green fluorescent cells, indicating that these two kinds of labeled cells were mixed in the small spots and that their localization was not separated well. The area near the surface had a higher cell density and contained two kinds of labeled cells. The inside area had a lower cell density and mainly contained green fluorescent cells. To characterize the labeled peritoneal cells, we performed fluorescence immunohistochemistry against a thin section of another cell aggregate using an anti-F4/80 antibody against the F4/80 receptor, which is typically considered a marker of mature macrophages. As shown in Fig. 7E, some positive cells stained with anti-F4/80 antibody were observed. In this section, a larger number of peritoneal cells labeled with thiol-OS/Rho showing red fluorescence were observed than labeled HeLa cells showing green fluorescence. As shown in the merged image, cells with various patterns of 4 kinds of fluorescence were observed. The majority of peritoneal cells labeled with thiol-OS/Rho were not stained with anti-F4/80 antibody (Fig. 7E, red arrowheads). These F4/80-negative peritoneal cells clearly had their nuclei stained with DAPI and were the main cells composing this cell aggregate. Many cells were stained with anti-F4/80 antibody and showed both red and green fluorescence (Fig. 7E, orange arrowheads). These cells indicated an association between F4/80-positive macrophages labeled with thiol-OS/Rho and labeled HeLa cells. Additionally, some cells were stained with anti-F4/80 antibody and showed green fluorescence (Fig. 7E, purple arrowheads) but not red fluorescence. These cells indicated an association of thiol-OS/Rho-unlabeled F4/80-positive macrophages with labeled HeLa cells. Some cells showing only fluorescence derived from the anti-F4/80 antibody (Fig. 7E, cyan arrowhead) were observed, indicating the involvement of thiol-OS/Rho-unlabeled F4/80-positive macrophages in the formation of this cell aggregate. Cells showing red and green fluorescence (Fig. 7E, yellow arrowhead) were observed, indicating F4/80-negative macrophages or other types of cells labeled with thiol-OS/Rho associated with HeLa cells. Therefore, at least 4 kinds of cells, including macrophages, were associated with labeled HeLa cells in this cell aggregate.

**Fig. 8:**
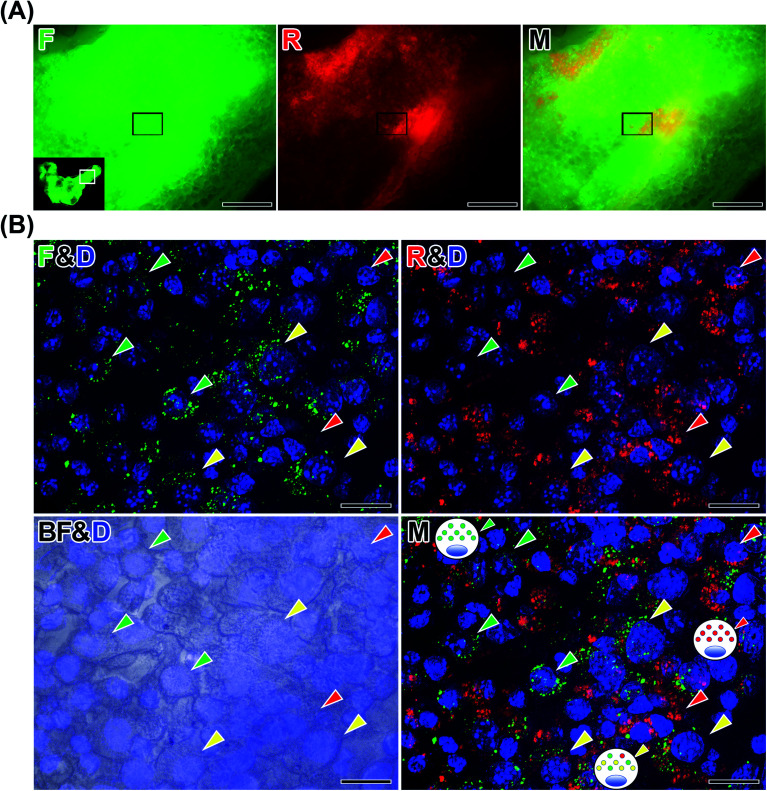
Detection of endosomal barcoded cells. The cultured J774A.1 cells were labeled with OS/Flu-PEI-2.0k and thiol-OS/RhoB showing barcoded endosomes *in vitro*. Thirty minutes before the injection of *in vitro*-labeled J774A.1 cells, we injected thiol-OS/Rho to label peritoneal macrophages. After 3 h, an aggregate was collected from the peritoneal cavity, stained with DAPI and observed on fluorescence microscopy. (A) At low magnification, the aggregate showed green and red fluorescence with different patterns. Scale bars: 200 μm. (B) At higher magnification, cells showing just red fluorescent endosomes (red arrowheads) and cells showing just green fluorescent endosomes (green arrowheads) were observed. The merged image revealed endosomal barcoded cells (yellow arrowhead) showing both red and green fluorescent endosomes. Scale bars: 20 μm.

Next, we performed *ex vivo* fluorescence imaging of an aggregate derived from a mouse injected with *in vitro*-labeled J774A.1 cells with OS/Flu-PEI2.0k and thiol-OS/Rho. Same as the case of HeLa cells, the mouse was injected thiol-OS/Rho to label peritoneal cells *in situ* before the injection of labeled J774A.1 cells. The *in vitro*-labeled J774A.1 cells also showed barcoded endosomes as shown in Fig. 4A2, and we tried to detect them. As shown in Fig. 8A, the aggregate showed both green and red fluorescence on fluorescence microscopy at low magnification. But the shape and fluorescence pattern were different from those of HeLa cells. The merged image showed green or red fluorescence dominant parts. The boundary between the area of strong red fluorescence and the area of strong green fluorescence was observed at high magnification (Fig. 8B). The cells in the aggregate showed green or red fluorescent endosomes. Many cells showed just green or red endosomes, but some cells showed both green and red fluorescent endosomes. The cells showing just green fluorescence indicated labeled J774A.1 cells treated with OS/Flu-PEI2.0k and thiol-OS/Rho and was just labeled with OS/Flu-PEI2.0k, but could not take up thiol-OS/Rho very well. The cells showing just red fluorescence indicated labeled peritoneal cells with thiol-OS/Rho. The cells showing both green and red fluorescent endosomes indicated labeled J774A.1 cells treated with and that could take up both OS/Flu-PEI2.0k and thiol-OS/Rho. These findings demonstrated successful detection of endosomal barcoded cells in the tissue collected from the body. The J774A.1 cells with barcoded endosomes would have different functions from the cells labeled with only OS/PEIs. It is important to understand their dynamics in the body and their significance of endosomal barcoded cells.

We conducted *in vivo* imaging of *in vitro*-labeled cells using thiol-OS/Flu-PEIs combined with *in situ*-labeled cells, followed by various microscopic observations including tissue clearing and immunohistochemistry. The fluorescence of *in vitro*- and *in situ*-labeled cells in cell aggregates was detected in *in vivo* imaging as well as in microscopy after treatment, such as fixation, tissue clearing, and frozen sectioning. These results demonstrated that cell labeling using OS/Flu-PEI was a very effective and useful method to seamlessly observe cells from macroscopic imaging *in vivo* to microscopic imaging *in vitro*. Transgene labeling of fluorescent proteins such as green fluorescent proteins has been widely applied to microscopic imaging *in vitro* to observe their expression and movement. In rare cases, overexpression of fluorescent proteins can induce apoptosis.^72^ The analysis of cell–nanoparticle interactions, including cell activity assays, provided appropriate conditions for NPs to label cells without cell death. The photointensity and photostability of fluorescent thiol-OS were superior to those of fluorescent dyes. Active cell labeling using OS/Flu-PEIs was simple, safe, and effective. Moreover, thiol-OS/Flu could serve as a multimodal probe for X-ray CT and MRI for multimodal imaging.^73–75^ We could not find labeled HeLa cells in the peritoneal cavity in optical imaging without the preinjection of thiol-OS/Rho. It would be possible to clarify the location and pathway of labeled HeLa cells lost in the peritoneal cavity by using other modalities. Labeled cells with multimodal OS/Flu-PEI would provide new potential for seamless observation among various imaging modalities as well as consecutive imaging macroscopic imaging *in vivo* to microscopic imaging *in vitro*.

We demonstrated peritoneal cell aggregates composed of labeled HeLa cells and macrophages labeled *in situ*. From another point of view, these findings indicated the immune response of activated macrophages against peritoneal metastatic cancer cells. Because thiol-OS increased the activity of macrophage cell lines *in vitro*, cell aggregates involving labeled HeLa cells might be formed by activated macrophages. For peritoneal metastasis, ovarian cancers (OCs), as well as gastrointestinal malignancies, including colorectal, gastric, and pancreatic cancers, are known to form multicellular aggregates or spheroids when they metastasize into the peritoneal cavity of advanced cancer patients. It was reported that the formation of multicellular spheroids was contributed by M2-like tumor-associated macrophages (TAMs).^76^ The ID8 OC cells expressing mCherry fluorescent protein were injected into mice intraperitoneally and formed multicellular spheroids containing CD68^+^ macrophages expressing green fluorescent protein. In the spheroids, OC cells surrounded the center-located macrophages. In Fig. 7D1 and 7D2, the cell aggregates exhibited some spots of fluorescence from labeled peritoneal macrophages surrounded by labeled HeLa cells. Our model of *in vivo* imaging of *in vitro*-labeled cells combined with *in situ*-labeled cells using NPs confirmed similarities with the model of OC peritoneal metastasis using fluorescent proteins. The biological significance of the cell aggregates on tumor progression in the peritoneal cavity in our model is under investigation. Fluorescent and multimodal thiol-organosilica NPs can be surface functionalized with PEI and applied to cell labeling for various applications. PEI surface functionalization of multimodal organosilica NPs and their cell labeling would open up the possibility for seamless imaging among various modalities, providing higher sensitivity, temporal and spatial resolution, and 3-D images and lead to innovative nanomedicine.

## Conclusions

In this study, we prepared and characterized 4 kinds of OS/Flu-PEI composed of different MW PEIs (1.3k, 2.0k, 25k, and 750k), which have individual physicochemical properties, including *ζ* potentials and aggregation-redispersion phenomena. We proposed their surface structure models based on the dependence of the PEI MW, *i.e.*, OS/Flu-PEI1.3k is a brush type; OS/Flu-PEI2.0k is a bent brush type; OS/Flu-PEI25k is a bent lie-down type; and OS/Flu-PEI750k is a coiled type. The clarification and understanding of the surface structure of particles are very important to control cell–nanoparticle interactions. OS/Flu-PEIs enabled efficient cell labeling of cancer and macrophage cell lines, but PEI-resistant macrophages were identified. The labeling ratio and labeling intensity were dependent on both the cell types and the PEI MW of each OS/Flu-PEI. The evaluation of intracellular labeling of OS/Flu-PEIs using simultaneous dual-particle administration identified endosomal barcoded cells indicating the differential intraendosomal sorting of the particles depending on particle surface structure at the single-cell level. There could be unknown sorting systems that distinguish and transfer NPs to each endosome depending on their surface structure. The cell activity assay revealed characteristic cytotoxicities and cell responses to OS/Flu-PEIs depending on the PEI MW, NP concentrations, and cell type. Two macrophage cell lines showed NP concentration-dependent alteration in mitochondrial activity against not only thiol-OS/Flu but also OS/Flu-PEIs. We conducted *in vivo* imaging of HeLa cells labeled *in vitro* with OS/Flu-PEI2.0k combined with *in situ* labeling of peritoneal cells using thiol-OS/Rho in the mouse intraperitoneal cavity. *In vitro*-labeled HeLa cells were detected and identified in multicellular aggregates *in vivo*, and the aggregates revealed the association of labeled HeLa cells with *in situ*-labeled intraperitoneal cells, including macrophages, in *ex vivo* cleared tissue and tissue sections. *In vitro*-labeled J774A.1 cells with OS/Flu-PEI2.0k and thiol-OS/were also injected intraperitoneally and detected and identified as endosomal barcoded cells in the aggregates. We showed a model of *in vivo* imaging of *in vitro*-labeled cells and *in vitro*-barcoded cells combined with *in situ*-labeled cells. *In vitro* cell labeling and barcoding using NPs provide a new generation of labeled cell imaging following GFP and fluorescent dye. The applications of multimodal NPs and combinations with well-developed cell labels, such as GFP, promise further expansion of cell imaging. Recent studies have reported the control of cell function,^77,78^ differentiation,^79–82^ or macrophage polarization^83,84^ using NPs. These cellular controls using NPs evolve into cell-based therapy. Cells labeled with NPs that possess functions such as multimodal imaging and cellular control could be developed for diagnostic imaging, therapy, and then cell-based nanotheranostics.

## Experimental

### Chemicals and reagents

3-Mercaptopropyltrimethoxysilane (MPMS), 3-aminopropyltrimethoxysilane (APS), fluorescein isothiocyanate isomer (FITC), rhodamine B, bisbenzimide H 33342 trihydrochloride (Hoechst 33342) and branched polyethyleneimine solution [MW = 1.3k (PEI1.3k), 2.0k (PEI2.0k), 25k (PEI25k), and 750k (PEI750k)] were purchased from Sigma-Aldrich Chemical Co. (St. Louis, MO, USA). Ammonium hydroxide (NH_4_OH, 28%) and ethanol were purchased from Kishida Chemical (Osaka, Japan). All other reagents and solvents were of analytical grade.

### Preparation of the fluorescent thiol-organosilica nanoparticles surface functionalized with PEI (OS/Flu-PEIs)

Thiol-OS/Flu and thiol-OS/were prepared using a one-pot procedure as described previously.^18^ Briefly, thiol-OS/Flu was prepared using APS–FITC conjugates. The conjugate was prepared by gently stirring a mixture of 250 mM APS and 250 mM FITC for 1 h. The solution of APS–FITC conjugates was mixed with a mixture of MPMS and 27% NH_4_OH. Thiol-OS/ was prepared using a solution mixture containing rhodamine B, MPMS and 27% NH_4_OH. For biological experiments, 1 mg mL^−1^ thiol-OS/Flu or thiol-OS/was treated with 10% PEI1.3k, PEI2.0k, PEI25k, or PEI750k in distilled water and incubated with mixing for 1 day at 37 °C. For evaluation of the aggregation-redispersion phenomenon, NPs were treated with 10% to 0.000001% PEIs in distilled water. After incubation, the mixture was centrifuged (20 000×*g*, 4 °C, 20 min) to remove the unbound reagent, and the pellets were resuspended with distilled water (D.W.) and sonicated and washed 3 times with distilled water.

### Dynamic light scattering (DLS)

DLS was carried out to analyze the size distribution and *ζ* potential of NPs using a DelsaMax PRO light scattering analyzer (Beckman Coulter, Brea, CA, USA) at 20 °C. Analyte thiol-OS/Flu and OS/Flu-PEIs treated with 10% to 1 × 10^−6^% PEIs were prepared by dispersing with distilled water (0.2 mg mL^−1^), and more than 3 parallel samples were measured after sonication.

### Electron microscopic observation

High resolution images of thiol-OS/Flu and thiol-OS/Rho were obtained using a JEM-F200 electron microscope (JEOL Ltd., Tokyo, Japan). Thiol-OS/Flu and OS/Flu-PEIs treated with 10% PEIs were dried and fixed on a 400 mesh copper grid (Nisshin EM Co., Tokyo, Japan) coated with polyvinyl formal. The scanning transmission electron microscopy (STEM) and scanning electron microscopy (SEM) images of the NPs were obtained using a Quanta 3D FEG electron microscope (FEI Company, Hillsboro, Oregon, USA). The images were analyzed with Image-Pro Plus 6.1J software (Media Cybernetics, Inc., Rockville, Maryland, USA).

### Thermal gravimetric analysis (TGA)

TGA of NPs was performed using a STA7200 thermal analysis system (Hitachi, Tokyo, Japan) in the temperature range of 30–800 °C with a heating rate of 10 °C min^−1^ under a 200 mL min^−1^ flow of nitrogen gas. The content rate of the organic component of OS/Flu-PEI treated with 10% PEIs was estimated from the difference between the weight at 300 °C and that at 800 °C.

### Cell activity assay

HeLa cells were maintained in DMED medium, and J774A.1 and RAW 264.7 cells were maintained in RPMI1640 medium. All cell culture media contained 10% fetal bovine serum, 100 U mL^−1^ penicillin and 100 μg mL^−1^ streptomycin. The cell activities of these cells were evaluated using the WST-1 (2-(2-methoxy-4-nitrophenyl)-3-(4-nitrophenyl)-5-(2,4-disulfophenyl)-2*H*-tetrazolium) cell proliferation assay reagent (Dojin Chemical Co., Kumamoto, Japan). The cells were seeded at a density of 2.0 × 10^4^ cells per well for HeLa cells and 4.0 × 10^4^ cells per well for J774A.1 and RAW 264.7 cells in 96-well plates 24 h before NP treatment. The cells were incubated with 0–1000 μg mL^−1^ thiol-OS/Flu and OS/Flu-PEI treated with 10% PEIs or left untreated in cell medium for 1 day at 37 °C. The absorbance of the NP solution was measured for each well immediately after the addition of the WST-1 reagent and after incubation at 37 °C. To obtain only signals derived from the formazan dye (produced by viable cells), the former signal was subtracted from the latter.

### Fluorescence microscopic observation

HeLa, MCF-7, RAW 264.7, and J774A.1 cells at a density of 2.0 × 10^4^ cells per well were seeded in 96-well plates. Mouse peritoneal macrophage cells from exudate from ICR mice at a density of 8.0 × 10^4^ cells per well were seeded. After treatment with medium containing thiol-OS/Flu or OS/Flu-PEI (final concentration: 10 μg mL^−1^) overnight, the cells were stained with Hoechst 33342 and observed on an IN Cell Analyzer 2000 (GE Healthcare, Chicago, IL) with excitation filters at passbands of 300–400 nm and 470–510 nm and emission filters at passbands of 405–505 nm and 489–561 nm for Hoechst 33342 and thiol-OS/Flu and OS/Flu-PEIs, respectively.

### Fluorescence microscopic imaging of single cells using dual particle administration

HeLa and J774A.1 cells were incubated (2 × 10^4^ per well in 96-well plates (Iwaki, Tokyo, Japan)) applying dual particles composed of thiol-OS and/or OS-PEI treated with 10% PEI. The cells were treated with a medium containing both thiol-OS/Flu and thiol-OS/Rho, both OS/Flu-PEI2.0k and thiol-OS/Rho, or both OS/Flu-PEI2.0k and OS/Rho-PEI750k (each particle final concentration: 10 μg mL^−1^). All of the particles in the medium were well suspended using sonication before the treatments. After 24 h, the cells were fixed with 1% (w/v) paraformaldehyde in PBS for 20 min. All images were obtained using a BZ-X800 microscope (Keyence, Osaka, Japan) and processed to create a full focus image from Z-stacks using BZ-X800 analyzer software (Keyence, Osaka, Japan).

### Flow cytometric analysis

A total of 2.0 × 10^5^ mouse peritoneal macrophages exudated from ICR mice, HeLa cells, and MCF-7 cells and 2.5 × 10^5^ J774A.1 cells were plated on 35 mm dishes and incubated at 37 °C for 1 day. The cells were treated with a medium containing thiol-OS/Flu or OS/Flu-PEI treated with 10% PEI (final concentration: 10 μg mL^−1^) for 6 h. All of the NPs in the medium were well suspended using sonication before the treatments. After incubation, all the cells were retrieved from the culture dish and subjected to flow cytometry. Flow cytometric analysis was performed using a FACSCalibur™ flow cytometer with Cell Quest software (Becton Dickinson, San Jose, USA) and 488 nm excitation lasers. Fluorescence was detected on the FL1 channel (530/30 nm bandpass filter). A total of 5000 cells were analyzed in each replicate. To evaluate mitochondrial conditions, cells treated with NPs were stained with tetramethylrhodamine ethyl ester perchlorate (TMRE, Invitrogen, Eugene, OR, USA) and subjected to flow cytometric analysis. Fluorescence was detected on the FL2 channel (filter with passband 543–627 nm).

### *In vivo* imaging of *in vitro*-labeled HeLa cells combined with *in situ*-labeled peritoneal cells

All experiments using animals in this study were approved by the Institutional Animal Care and Use Committee and carried out according to the Guidelines for Animal Experimentation of Yamaguchi University School of Medicine. Male ICR mice (from 8 to 9 weeks of age) obtained from Japan SLC (Shizuoka, Japan) were used in this study. HeLa cells (2 × 10^6^) or J774A.1 cells (2 × 10^6^) were plated on a 100 mm dish and incubated at 37 °C for 1 day. HeLa cells were treated overnight with a medium containing OS/Flu-PEI2.0k treated with 10% PEI (with a final concentration of 10 μg mL^−1^). J774A.1 cells were applied with dual particles and treated overnight with a medium containing thiol-OS/and OS/Flu-PEI2.0k treated with thiol-OS/and OS/Flu-PEI2.0k (each particle final concentration: 10 μg mL^−1^). All of the medium containing NPs was well suspended using sonication before the treatments. After incubation, all cells were retrieved from the culture dish, and cell suspensions containing labeled HeLa cells or J774A.1 cells (0.1 mL) were prepared. To label peritoneal cells *in situ*, mice were intraperitoneally administered a suspension containing 0.5 mg of thiol-OS/Rho before the injection of labeled cell suspension. The labeled cell suspension was intraperitoneally administered to mice. After 3 h, the mice were euthanized, and the objects showing fluorescence in the abdominal cavity were observed and isolated under observation on a Nikon AZ100 multizoom microscope (Kanagawa, Japan) and fixed with 4% (w/v) paraformaldehyde in PBS at 4 °C overnight. *Ex vivo* imaging of the objects showed fluorescence with and without tissue clearing using ScaleCUBIC-1 ^85^ and was conducted using a BZ-X800 microscope. Another object was frozen in the OCT compound (Sakura Finetek Japan, Tokyo, Japan) and cut into 6 μm for immunostaining or 20 μm sections for single cell observation. For immunostaining, the tissue sections of 6 μm were treated with a 100-fold diluted anti-F4/80 antibody (GeneTex, Inc., Los Angeles, CA, USA) at 4 °C overnight, washed with PBS, and treated with a secondary antibody (100-fold diluted Cy™5 AffiniPure Goat Anti-Rat IgG (H + L) (Jackson ImmunoResearch Inc., West Grove, PA, USA) at room temperature for 1 h. After nuclear staining with DAPI, all images were obtained using a BZ-X800 microscope.

## Conflicts of interest

There are no conflicts to declare.

## Supplementary Material

NA-004-D1NA00839K-s001

NA-004-D1NA00839K-s002

NA-004-D1NA00839K-s003

NA-004-D1NA00839K-s004

## References

[cit1] Shimomura O., Johnson F. H., Saiga Y. (1962). J. Cell. Comp. Physiol..

[cit2] Rodriguez E. A., Campbell R. E., Lin J. Y., Lin M. Z., Miyawaki A., Palmer A. E., Shu X., Zhang J., Tsien R. Y. (2017). Trends Biochem. Sci..

[cit3] Horan P. K., Slezak S. E. (1989). Nature.

[cit4] Hendrikx J. V. P. J., Martens C., Hagenbeek A., Keij J. (1996). Exp. Hematol..

[cit5] Sipkins D. A., Wei X., Wu J. W., Runnels J. M., Côté D., Means T. K., Luster A. D., Scadden D. T., Lin C. P. (2005). Nature.

[cit6] Lewandowski D., Barroca V., Ducongé F., Bayer J., Van Nhieu J. T., Pestourie C., Fouchet P., Tavitian B., Roméo P. H. (2010). Blood.

[cit7] Zhao W., Schafer S., Choi J., Yamanaka Y. J., Lombardi M. L., Bose S., Carlson A. L., Phillips J. A., Teo W., Droujinine I. A., Cui C. H., Jain R. K., Lammerding J., Love J. C., Lin C. P., Sarkar D., Karnik R., Karp J. M. (2011). Nat. Nanotechnol..

[cit8] Ahrens E. T., Bulte J. W. M. (2013). Nat. Rev. Immunol..

[cit9] Bernsen M. R., Guenoun J., Van Tiel S. T., Krestin G. P. (2015). Br. J. Radiol..

[cit10] Deng S. L., Li Y. Q., Zhao G. (2018). Chin. Med. J..

[cit11] Chandrasekaran R., Madheswaran T., Tharmalingam N., Bose R. J., Park H., Ha D. H. (2021). Drug Discovery Today.

[cit12] Bouché M., Hsu J. C., Dong Y. C., Kim J., Taing K., Cormode D. P. (2020). Bioconjugate Chem..

[cit13] Nakamura M. , Organosilica Nanoparticles and Medical Imaging, Elsevier Inc., 1st edn, 2018, vol. 44 10.1016/bs.enz.2018.08.002 30360813

[cit14] Nakamura M. , Nanostructured Oxides, ed. C. S. S. R. Kumar, Wiley-VCH Verlag GmbH & Co. KGaA, Germany, 2010, pp. 109–161

[cit15] Mochizuki C., Nakamura J., Nakamura M. (2021). Biomedicines.

[cit16] Nakamura M., Miyamoto K., Hayashi K., Awaad A., Ochiai M., Ishimura K. (2013). Nanomed.: Nanotechnol. Biol. Med..

[cit17] Nakamura M., Hayashi K., Nakano M., Kanadani T., Miyamoto K., Kori T., Horikawa K. (2015). ACS Nano.

[cit18] Nakamura M., Ozaki S., Abe M., Doi H., Matsumoto T., Ishimura K. (2010). Colloids Surf., B.

[cit19] Nakamura M., Ishimura K. (2010). Adv. Sci. Lett..

[cit20] Kim H., Röth D., Isoe Y., Hayashi K., Mochizuki C., Kalkum M., Nakamura M. (2021). Colloids Surf., B.

[cit21] Choudhury C. K., Roy S. (2013). Soft Matter.

[cit22] Milner S. T. (1988). Epl.

[cit23] Kenworthy A. K., Simon S. A., McIntosh T. J. (1995). Biophys. J..

[cit24] Nakamura M., Ishimura K. (2007). J. Phys. Chem. C.

[cit25] Nakamura M., Ishimura K. (2008). Langmuir.

[cit26] Doura T., Nishio T., Tamanoi F., Nakamura M. (2019). J. Mater. Res..

[cit27] Nakamura M., Ishimura K. (2008). Langmuir.

[cit28] Sehgal A., Lalatonne Y., Berret J. F., Morvan M. (2005). Langmuir.

[cit29] Naka K., Tanaka H., Chujo Y. (2007). Polym. J..

[cit30] Chanteau B., Fresnais J., Berret J. F. (2009). Langmuir.

[cit31] Awaad A., Nakamura M., Ishimura K. (2012). Nanomed.: Nanotechnol. Biol. Med..

[cit32] Barata-Antunes C., Alves R., Talaia G., Casal M., Gerós H., Mans R., Paiva S. (2021). Comput. Struct. Biotechnol. J..

[cit33] Hui Y., Yi X., Wibowo D., Yang G., Middelberg A. P. J., Gao H., Zhao C. X. (2020). Sci. Adv..

[cit34] Dadhich R., Kapoor S. (2020). J. Membr. Biol..

[cit35] Münz C. (2020). Semin. Cancer Biol..

[cit36] Hinze C., Boucrot E. (2018). J. Cell Sci..

[cit37] Manzanares D., Ceña V. (2020). Pharmaceutics.

[cit38] Yang N., Shen H. M. (2020). Int. J. Biol. Sci..

[cit39] Bayati A., Kumar R., Francis V., McPherson P. S. (2021). J. Biol. Chem..

[cit40] Poduri R., Joshi G., Jagadeesh G. (2020). Cell. Signal..

[cit41] Bayguinov P. O., Fisher M. R., Fitzpatrick J. A. J. (2020). J. Biol. Chem..

[cit42] Brown E., Verkade P. (2010). Protoplasma.

[cit43] Sochacki K. A., Taraska J. W. (2021). Curr. Opin. Cell Biol..

[cit44] Nakamura M., Hayashi K., Nakamura J., Mochizuki C., Murakami T., Miki H., Ozaki S., Abe M. (2020). Chem. Mater..

[cit45] Rai R., Alwani S., Badea I. (2019). Polymers.

[cit46] Haladjova E., Rangelov S., Tsvetanov C. (2020). Polymers.

[cit47] Höbel S., Aigner A. (2013). Wiley Interdiscip. Rev.: Nanomed. Nanobiotechnol..

[cit48] Basarkar A., Singh J. (2007). Int. J. Nanomed..

[cit49] Fischer D., Bieber T., Li Y., Elsässer H. P., Kissel T. (1999). Pharm. Res..

[cit50] Okon E. U., Hammed G., Abu P., Wafa E., Abraham O., Case N., Henry E. (2014). Int. J. Innovation Appl. Stud..

[cit51] Monnery B. D., Wright M., Cavill R., Hoogenboom R., Shaunak S., Steinke J. H. G., Thanou M. (2017). Int. J. Pharm..

[cit52] Xia T., Kovochich M., Liong M., Meng H., Kabehie S., George S., Zink J. I., Nel A. E. (2009). ACS Nano.

[cit53] Feng Q., Liu Y., Huang J., Chen K., Huang J., Xiao K. (2018). Sci. Rep..

[cit54] Hong S., Bielinska A. U., Mecke A., Keszler B., Beals J. L., Shi X., Balogh L., Orr B. G., Baker J. R., Banaszak Holl M. M. (2004). Bioconjugate Chem..

[cit55] Hong S., Leroueil P. R., Janus E. K., Peters J. L., Kober M. M., Islam M. T., Orr B. G., Baker J. R., Banaszak Holl M. M. (2006). Bioconjugate Chem..

[cit56] Leroueil P. R., Berry S. A., Duthie K., Han G., Rotello V. M., McNerny D. Q., Baker J. R., Orr B. G., Holl M. M. B. (2008). Nano Lett..

[cit57] Liu Y., Liu J. (2020). Langmuir.

[cit58] Hall A., Lächelt U., Bartek J., Wagner E., Moghimi S. M. (2017). Mol. Ther..

[cit59] Hall A., Larsen A. K., Parhamifar L., Meyle K. D., Wu L. P., Moghimi S. M. (2013). Biochim. Biophys. Acta, Bioenerg..

[cit60] Benjaminsen R. V., Mattebjerg M. A., Henriksen J. R., Moghimi S. M., Andresen T. L. (2013). Mol. Ther..

[cit61] Xia T., Kovochich M., Liong M., Zink J. I., Nel A. E. (2008). ACS Nano.

[cit62] Liang W. L., Xiao L., Gu H. W., Li X. J., Li Y. S., Zhang W. K., Bin Tang H. (2019). Int. J. Nanomed..

[cit63] Park E. J., Choi D. H., Kim Y., Lee E. W., Song J., Cho M. H., Kim J. H., Kim S. W. (2014). Toxicol. In Vitro.

[cit64] Carlson C., Hussein S. M., Schrand A. M., Braydich-Stolle L. K., Hess K. L., Jones R. L., Schlager J. J. (2008). J. Phys. Chem. B.

[cit65] Li J., Zhang Y., Xiao Q., Tian F., Liu X., Li R., Zhao G., Jiang F., Liu Y. (2011). J. Hazard. Mater..

[cit66] Khan M. I., Mohammad A., Patil G., Naqvi S. A. H., Chauhan L. K. S., Ahmad I. (2012). Biomaterials.

[cit67] Piao M. J., Kang K. A., Lee I. K., Kim H. S., Kim S., Choi J. Y., Choi J., Hyun J. W. (2011). Toxicol. Lett..

[cit68] Hsin Y. H., Chen C. F., Huang S., Shih T. S., Lai P. S., Chueh P. J. (2008). Toxicol. Lett..

[cit69] Huang Z., Yang Y., Jiang Y., Shao J., Sun X., Chen J., Dong L., Zhang J. (2013). Biomaterials.

[cit70] Mulens-Arias V., Rojas J. M., Pérez-Yagüe S., Morales M. P., Barber D. F. (2015). Biomaterials.

[cit71] Urbanska A. M., Khanin R., Alidori S., Wong S., Mello B. P., Almeida B. A., Chen F., Ma K., Turker M. Z., Korontsvit T., Scheinberg D. A., Zanzonico P. B., Wiesner U., Bradbury M. S., Quinn T. P., McDevitt M. R. (2020). Cancer Biother.Radiopharm..

[cit72] Liu H. S., Jan M. S., Chou C. K., Chen P. H., Ke N. J. (1999). Biochem. Biophys. Res. Commun..

[cit73] Nakamura M., Awaad A., Hayashi K., Ochiai K., Ishimura K. (2012). Chem. Mater..

[cit74] Nakamura M., Hayashi K., Kubo H., Kanadani T., Harada M., Yogo T. (2017). J. Colloid Interface Sci..

[cit75] Nakamura M., Hayashi K., Kubo H., Harada M., Izumi K., Tsuruo Y., Yogo T. (2017). Sci. Rep..

[cit76] Yin M., Li X., Tan S., Zhou H. J., Ji W., Bellone S., Xu X., Zhang H., Santin A. D., Lou G., Min W. (2016). J. Clin. Invest..

[cit77] Zanganeh S., Hutter G., Spitler R., Lenkov O., Mahmoudi M., Shaw A., Pajarinen J. S., Nejadnik H., Goodman S., Moseley M., Coussens L. M., Daldrup-Link H. E. (2016). Nat. Nanotechnol..

[cit78] Genchi G. G., Marino A., Grillone A., Pezzini I., Ciofani G. (2017). Adv. Healthcare Mater..

[cit79] Kang H., Jung H. J., Wong D. S. H., Kim S. K., Lin S., Chan K. F., Zhang L., Li G., Dravid V. P., Bian L. (2018). J. Am.
Chem. Soc..

[cit80] Kang H., Jung H. J., Kim S. K., Wong D. S. H., Lin S., Li G., Dravid V. P., Bian L. (2018). ACS Nano.

[cit81] Yun S., Shin T. H., Lee J. H., Cho M. H., Kim I. S., Kim J. W., Jung K., Lee I. S., Cheon J., Park K. I. (2018). Nano Lett..

[cit82] Khatua C., Min S., Jung H. J., Jung H. J., Jung H. J., Shin J. E., Li N., Jun I., Liu H. W., Bae G., Choi H., Ko M. J., Jeon Y. S., Kim Y. J., Lee J., Ko M., Shim G., Shin H., Lee S., Chung S., Chung S., Chung S., Kim Y. K., Kim Y. K., Song J. J., Dravid V. P., Dravid V. P., Dravid V. P., Kang H. (2020). Nano Lett..

[cit83] Choi H., Bae G., Khatua C., Min S., Jung H. J., Li N., Jun I., Liu H. W., Cho Y., Na K. H., Ko M., Shin H., Kim Y. H., Chung S., Song J. J., Dravid V. P., Kang H. (2020). Adv. Funct. Mater..

[cit84] Kang H., Zhang K., Wong D. S. H., Han F., Li B., Bian L. (2018). Biomaterials.

[cit85] Susaki E. A., Tainaka K., Perrin D., Kishino F., Tawara T., Watanabe T. M., Yokoyama C., Onoe H., Eguchi M., Yamaguchi S., Abe T., Kiyonari H., Shimizu Y., Miyawaki A., Yokota H., Ueda H. R. (2014). Cell.

